# Associations of Antenatal Corticosteroids With Neurodevelopment in Children Aged 27–30 Months: A Population‐Based Cohort Study

**DOI:** 10.1111/1471-0528.18101

**Published:** 2025-02-19

**Authors:** Emily M. Frier, Marius Lahti‐Pulkkinen, Chun Lin, Fabienne Decrue, Helga Zoega, Karel Allegaert, Jasper V. Been, David Burgner, Kate Duhig, Kristjana Einarsdóttir, Lani Florian, Abigail Fraser, Mika Gissler, Cynthia Gyamfi‐Bannerman, Lars Henning Pedersen, Jessica E. Miller, Ben W. Mol, Sarah R. Murray, Jane Norman, Devender Roberts, Ewoud Schuit, Ting Shi, Aziz Sheikh, Joshua P. Vogel, Rachael Wood, Emma McGoldrick, Bo Jacobsson, Eyal Krispin, Rebecca M. Reynolds, Sarah J. Stock, the Co‐OPT Collaboration, Karel Allegaert, Jasper Been, David Burgner, Kate Duhig, Kristjana Einarsdóttir, John Fahey, Lani Florian, Abigail Fraser, Mika Gissler, Cynthia Gyamfi‐Bannerman, Bo Jacobsson, Eyal Krispin, Stefan Kuhle, Marius Lahti‐Pulkkinen, Jessica Miller, Ben Mol, Sarah Murray, Jane Norman, Lars Henning Pedersen, Richard Riley, Devender Roberts, Ewoud Schuit, Aziz Sheikh, Ting Shi, Claire Tochel, Joshua Vogel, Rachael Wood, John Wright, Helga Zoega, Karel Allegaert, Jasper Been, David Burgner, Kate Duhig, Kristjana Einarsdóttir, John Fahey, Lani Florian, Abigail Fraser, Mika Gissler, Cynthia Gyamfi‐Bannerman, Bo Jacobsson, Eyal Krispin, Stefan Kuhle, Marius Lahti‐Pulkkinen, Jessica Miller, Ben Mol, Sarah Murray, Jane Norman, Lars Henning Pedersen, Richard Riley, Devender Roberts, Ewoud Schuit, Aziz Sheikh, Ting Shi, Claire Tochel, Joshua Vogel, Rachael Wood, John Wright, Helga Zoega

**Affiliations:** ^1^ MRC Centre for Reproductive Health The Queen's Medical Research Institute, The University of Edinburgh Edinburgh UK; ^2^ Department of Psychology and Logopedics, Faculty of Medicine University of Helsinki and Helsinki University Central Hospital Helsinki Finland; ^3^ Centre for Cardiovascular Science The Queen's Medical Research Institute, The University of Edinburgh Edinburgh UK; ^4^ Usher Institute, The University of Edinburgh Edinburgh UK; ^5^ Faculty of Medicine and Health School of Population Health, UNSW Sydney Sydney New South Wales Australia; ^6^ Centre of Public Health Sciences, Faculty of Medicine University of Iceland Reykjavik Iceland; ^7^ Department of Pharmaceutical and Pharmacological Sciences KU Leuven Leuven Belgium; ^8^ Department of Hospital Pharmacy Erasmus MC Rotterdam the Netherlands; ^9^ Department of Neonatal and Paediatric Intensive Care, Division of Neonatology, Erasmus MC Sophia Children's Hospital University Medical Centre Rotterdam Rotterdam the Netherlands; ^10^ Department of Obstetrics and Gynaecology, Erasmus MC Sophia Children's Hospital University Medical Centre Rotterdam Rotterdam the Netherlands; ^11^ Department of Paediatrics Melbourne University Parkville Victoria Australia; ^12^ Murdoch Children's Research Institute, Royal Children's Hospital Melbourne Victoria Australia; ^13^ Maternal and Fetal Health Research Centre University of Manchester Manchester UK; ^14^ Faculty of Health Sciences Curtin University Bentley Western Australia Australia; ^15^ Moray House School of Education and Sport University of Edinburgh Edinburgh UK; ^16^ Population Health Sciences Bristol Medical School and MRC Integrative Epidemiology Unit at the University of Bristol Bristol UK; ^17^ Department of Molecular Medicine and Surgery Karolinska Institutet Stockholm Sweden; ^18^ Knowledge Brokers, Finnish Institute for Health and Welfare Helsinki Finland; ^19^ Department of Obstetrics, Gynaecology, and Reproductive Sciences University of California San Diego, La Jolla California USA; ^20^ Department of Clinical Medicine Aarhus University Aarhus Denmark; ^21^ Department of Obstetrics and Gynaecology Aarhus University Hospital Aarhus Denmark; ^22^ Ritchie Centre Monash University Clayton Australia; ^23^ Aberdeen Centre for Women's Health Research, School of Medicine, Medical Sciences and Nutrition University of Aberdeen Aberdeen UK; ^24^ University of Nottingham Nottingham UK; ^25^ Family Health Division, Liverpool Women's Hospital Liverpool UK; ^26^ Department of Epidemiology & Health Economics, Julius Center for Health Sciences and Primary Care University Medical Center Utrecht, Utrecht University Utrecht the Netherlands; ^27^ Maternal, Child and Adolescent Health Program, Burnet Institute Melbourne Victoria Australia; ^28^ Public Health Scotland Edinburgh UK; ^29^ Department of Obstetrics and Gynecology Institute of Clinical Science, Sahlgrenska Academy, University of Gothenburg Gothenburg Sweden; ^30^ Department of Obstetrics and Gynecology Sahlgrenska University Hospital Region Västra Götaland, Gothenburg Sweden; ^31^ Maternal Fetal Care Center, Boston Children's Hospital Boston Massachusetts USA

**Keywords:** antenatal corticosteroids, neurodevelopment, preterm birth

## Abstract

**Objective:**

To examine the associations of antenatal corticosteroid (ACS) exposure with neurodevelopment in early childhood, and how these vary with gestational age at birth.

**Design:**

Population‐based cohort study.

**Setting:**

Scotland, UK.

**Population:**

285 637 singleton children born at 28–41 weeks' gestation, between 1st January 2011 and 31st December 2017, who underwent health reviews at 27–30 months of age.

**Methods:**

Logistic and linear regression analyses, stratified by gestation at birth (28–33, 34–36, 37–38 and 39–41 weeks' gestation), were used to evaluate the associations between ACS exposure and neurodevelopmental outcomes, and adjusted for maternal age, body mass index, diabetes, antenatal smoking, parity, neighbourhood deprivation, birth year, child sex and age at review.

**Main Outcome Measures:**

Practitioner‐identified concerns about any neurodevelopmental domain, and the average of five domain scores on neurodevelopmental milestones from the parent‐rated Ages and Stages Questionnaire (ASQ‐3).

**Results:**

After adjustment for covariates, ACS exposure was associated with reduced neurodevelopmental concerns in children born at 28–33 weeks' gestation (OR = 0.79, 95% CI = 0.62–0.999) and with increased neurodevelopmental concerns in children born at 34–36 weeks' gestation (OR = 1.11, 95% CI = 1.01–1.21). No independent associations emerged in children born at later gestations. ACS exposure was not associated with ASQ‐3 scores in any gestational age group.

**Conclusions:**

In early childhood, ACS exposure was associated with statistically significantly reduced neurodevelopmental concerns in children born at 28–33 weeks' gestation, and with statistically significantly increased neurodevelopment concerns in children born at 34–36 weeks' gestation. However, the effect sizes of these associations were small. No independent associations were found between ACS exposure and neurodevelopment in term‐born children.

## Abstract 2

**Linked article:** This article is commented on by Dude pp. 916–917 in this issue. To view this article visit https://doi.org/10.1111/1471‐0528.18136.

## 1 Introduction

Antenatal corticosteroids (ACS) are widely used before preterm birth (before 37 weeks' gestation) to reduce neonatal mortality, respiratory distress syndrome and intraventricular haemorrhage [1]. However, potential overuse of ACS is a concern [2], as up to half of all babies exposed to ACS are subsequently born at term (≥ 37 weeks' gestation) [3], when neonatal benefits are minimal [4]. Fetal overexposure to glucocorticoids may contribute to programming of disease later in life [5].

Evidence on long‐term neurodevelopmental outcomes associated with ACS exposure is conflicting. In a systematic review and meta‐analysis [6], two studies [7, 8] showed that ACS exposure was associated with reduced odds of neurodevelopmental impairment in children aged 18–22 months who had been born extremely preterm (defined as 22–27 weeks' gestation and/or birthweight of 401–1000 g). In contrast, in children born late preterm (34–36 weeks' gestation), a single study [9] found that ACS exposure was associated with higher risk of adverse neurodevelopmental outcomes. Follow up from the Antenatal Late Preterm Steroids Trial [10] reported similar neurodevelopmental outcomes in children aged 7 years who were exposed to ACS or placebo at 34–36 weeks' gestation [11].

Two cohort studies [12, 13] in the meta‐analysis [6] reported neurodevelopmental outcomes in ACS‐exposed term‐born children; risk of mental and behavioural disorders was increased [12], with more assessments for suspected neurocognitive disorders compared to term‐born children unexposed to ACS [13]. A subsequent observational study associated ACS exposure with an increased risk of psychological, developmental and neurosensory disorders in term‐born children, with no such associations in children born preterm [14]. In another population‐based study, ACS exposure was associated with poorer cognitive development in term‐born children [15]. Information on potential associations of ACS with milder neurodevelopmental impairment is sparse, particularly in term‐born children.

Better understanding of associations between ACS exposure and childhood neurodevelopment would inform clinical decision‐making and elucidate how effects of ACS vary by gestational age at birth, which is strongly associated with child neurodevelopment [16, 17, 18, 19]. This longitudinal population‐based study examined the associations of ACS exposure with early childhood neurodevelopment, and whether these varied with gestational age. Continuous and categorical neurodevelopmental assessments from population‐wide child health reviews at 27–30 months of age were used.

## 2 Methods

### 2.1 Study Population

Population‐based data from Scotland were utilised from the Co‐OPT (Consortium for the Study of Pregnancy Treatments) ACS cohort, described previously [2]. The Co‐OPT ACS cohort is an international database of 2.28 million births, containing information on ACS exposure and pregnancy and childhood outcomes [2]. From the Co‐OPT ACS cohort, all singleton children born in Scotland from 28 to 41 weeks' gestation, between 1st January 2011 and 31st December 2017, were identified from Maternity Inpatient and Day Case Scottish Morbidity Records 02 (SMR02). Births within these dates were included, since data on 27–30 month reviews, provided by the Child Health Surveillance Programme Pre‐School (CHSP‐PS) system, were available in the Co‐OPT ACS cohort from 2013 to 2019 [2]. Exclusion criteria included birth before 28 weeks' gestation and after 41 weeks' gestation, because high or low rates of ACS exposure in these children, respectively, would preclude meaningful comparisons between exposed and unexposed children.

Stillbirths, neonatal deaths or child deaths before 24 months of age (the minimum eligible age for 27–30 month review [20]) were excluded through record linkage of SMR02 with National Records of Scotland. Records of children who survived to at least 24 months were linked with 27–30 month review data in the CHSP‐PS system. Children with missing ACS exposure or 27–30 month review data were excluded.

The Scottish Child Health Programme (CHP) is provided by National Health Service Scotland to evaluate children's health, growth and development [21]. CHP reviews are undertaken at prespecified milestones by practitioners, typically health visitors (nurses or midwives trained in community public health nursing). The 27–30 month review aims to identify developmental delay [22] and is offered to all children in Scotland aged 24 to 35 months [20]. The CHSP‐PS system automatically invites children to scheduled reviews at the appropriate age [21].

Data sources, their validity and consistency, and data linkage processes were described previously [2]. Data quality assurance undertaken by Public Health Scotland reported high accuracy (91%) of hard‐coded variables in SMR02 [23], and high population representativeness of SMR02 delivery records (98% of all statutory registered births in Scotland [24]) and CHSP‐PS records (27–30 month review data available for 87%–92% of children eligible for review from 2013 to 2019 [25]).

The study is reported in accordance with STROBE guidelines [26] (Text S1).

### 2.2 ACS Exposure

The exposure was ACS, identified from the Antenatal Steroids field in SMR02 delivery records, where ACS exposure is coded as mutually exclusive categories of “ACS given”, “ACS not given”, “Not applicable (gestations of 36 weeks or over with no previous threat of delivery)” or “Not known whether given or not” [27]. Children coded as “ACS not given” or “Not applicable” were considered non‐ACS‐exposed. Children with ACS status “Not known whether given or not” were excluded.

During the time studied, clinical practice on ACS administration in Scotland was based on guidance from the Royal College of Obstetricians & Gynaecologists (published in 2010, archived in 2016) [28] and the National Institute for Health and Care Excellence (published in 2015) [29]. Table S1 provides information on these guidelines.

### 2.3 Outcomes

The outcomes were the presence of a practitioner‐identified concern about at least one neurodevelopmental domain, referred to as “practitioner concern about neurodevelopment”, and the continuous, parent‐assessed “Ages and Stages Questionnaire Third Edition (ASQ‐3) neurodevelopment score” [30, 31]. Table S2 provides detailed definitions of outcomes.

#### 2.3.1 Practitioner Concern About Neurodevelopment

Practitioner‐assessed developmental domains at the 27–30 month review included speech, language and communication, emotional/behavioural development, personal/social development, gross motor, fine motor, vision, hearing, and from 2017, problem‐solving (Table S3) [20]. Practitioners formed an overall judgement of child development in each domain based on a discussion with parents about the child's development and attainment of milestones, eliciting whether the parents had concerns regarding the child's development, the practitioner's own structured observation of the child, and/or results of the parent‐assessed ASQ‐3 questionnaire [21]. Practitioners recorded assessments of each domain as “no concern”, “concern newly suspected” or “concern already known”. A “practitioner concern about neurodevelopment” was identified if a newly suspected or a pre‐existing concern was recorded in any developmental domain assessed, except the problem‐solving domain, which was introduced to the 27–30 month review in 2017, and which was therefore excluded from this outcome to enable inclusion of reviews undertaken before and after 2017 (Table S2).

#### 2.3.2 ASQ‐3 Neurodevelopment Score

Continuous child neurodevelopment was assessed with the parent‐rated questionnaire, ASQ‐3 [30, 31], a widely used developmental screening tool, validated in multiple cultures [32, 33]. Since 2017, practitioners have asked parents to complete the ASQ‐3 at the 27–30 month review [20]. The ASQ‐3 is a series of age‐specific questionnaires on whether a child can complete developmental milestones across five domains: communication, gross motor, fine motor, problem‐solving and personal/social development. Each domain includes six questions, scored as 0 (child cannot yet complete this activity), 5 (child can sometimes complete this activity) or 10 (child has mastered this activity), yielding five subscale scores, ranging from 0 to 60 [30, 31]. The ASQ‐3 neurodevelopment score was the mean of ASQ‐3 scores in these five developmental domains (scored 0–60). ASQ subscale scores have been used to indicate neurodevelopmental delay [34, 35] and attainment of age‐specific developmental milestones [34, 35]; their mean has been used as a continuous index of neurodevelopment [36].

### 2.4 Covariates and Possible Confounders

A directed acyclic graph was used to identify possible mechanisms driving associations between ACS exposure and neurodevelopment, and potential confounding factors (Figure S1) [37]. The following covariates and possible confounders were included in regression analyses: maternal age at birth (years), parity (primiparous/multiparous), maternal body mass index (BMI, kg/m^2^) at first antenatal appointment (underweight (< 18.5)/normal weight (18.5–24.9)/overweight (25–29.9)/obese (≥ 30)), maternal smoking at first antenatal appointment (yes/no), maternal diabetes (yes/no), birth year, child sex (female/male) and neighbourhood deprivation (continuous variable derived from Scottish Index of Multiple Deprivation (SIMD) deciles [38]). The SIMD is an area‐based tool which identifies geographical deprivation areas across Scotland, by combining seven deprivation domains to assign an individual a SIMD ranking based on their neighbourhood of residence [38]. Child age at review was calculated based on birth date in SMR02 and review date in CHSP‐PS.

### 2.5 Statistical Analyses

Associations of maternal, pregnancy and child characteristics with ACS exposure and neurodevelopmental outcomes were assessed using *χ* ^2^ tests, *t*‐tests, and analyses of variance. ASQ‐3 neurodevelopment scores and child age were rank‐order normalised to account for skewness; these were thereafter converted into SD units, as were maternal age and neighbourhood deprivation.

Associations between ACS exposure and practitioner concerns about neurodevelopment were examined with logistic regression. Associations between ACS exposure and ASQ‐3 neurodevelopment scores were assessed with linear regression. In both linear and logistic regression analyses, one unadjusted model (Model 1) and two differentially adjusted models were used. Model 2 adjusted for child age and sex, which are fundamental covariates for evaluation of child neurodevelopment [39, 40]. Fully adjusted models (Model 3) adjusted for Model 2 covariates and for maternal age, BMI, smoking, parity, diabetes, birth year and neighbourhood deprivation. Interactions of ACS with child sex on the outcomes were also tested. Children with missing data on categorical covariates were included by classifying them into their own categories. We also conducted sensitivity analyses limited to each mother's first (or only) child born in the cohort to evaluate possible bias by partially dependent observations.

Results of logistic and linear regression models are presented as odds ratios and unstandardised regression coefficients, respectively, with 95% confidence intervals. Statistical significance was inferred from two‐sided significance tests with *p* ≤ 0.05.

Gestational age at birth was considered as a covariate, given its association with the exposure [12, 13, 14] and outcomes [16, 17, 18, 40], but as gestational age and ACS were multicollinear, analyses stratified for gestational age at birth instead. Categories included children born at 28–33, 34–36, 37–38, and 39–41 weeks' gestation. Children born preterm were categorised into these groups instead of the recommended World Health Organisation intervals [41] to enable evaluation of outcomes in children born late preterm.

Analyses were conducted using IBM SPSS Statistics 27.

### 2.6 Patient and Public Involvement

The research question was identified as a priority by the Co‐OPT Parental Advisory Board, a group which represents the interests of preterm and newborn babies and their families.

## 3 Results

**FIGURE 1:**
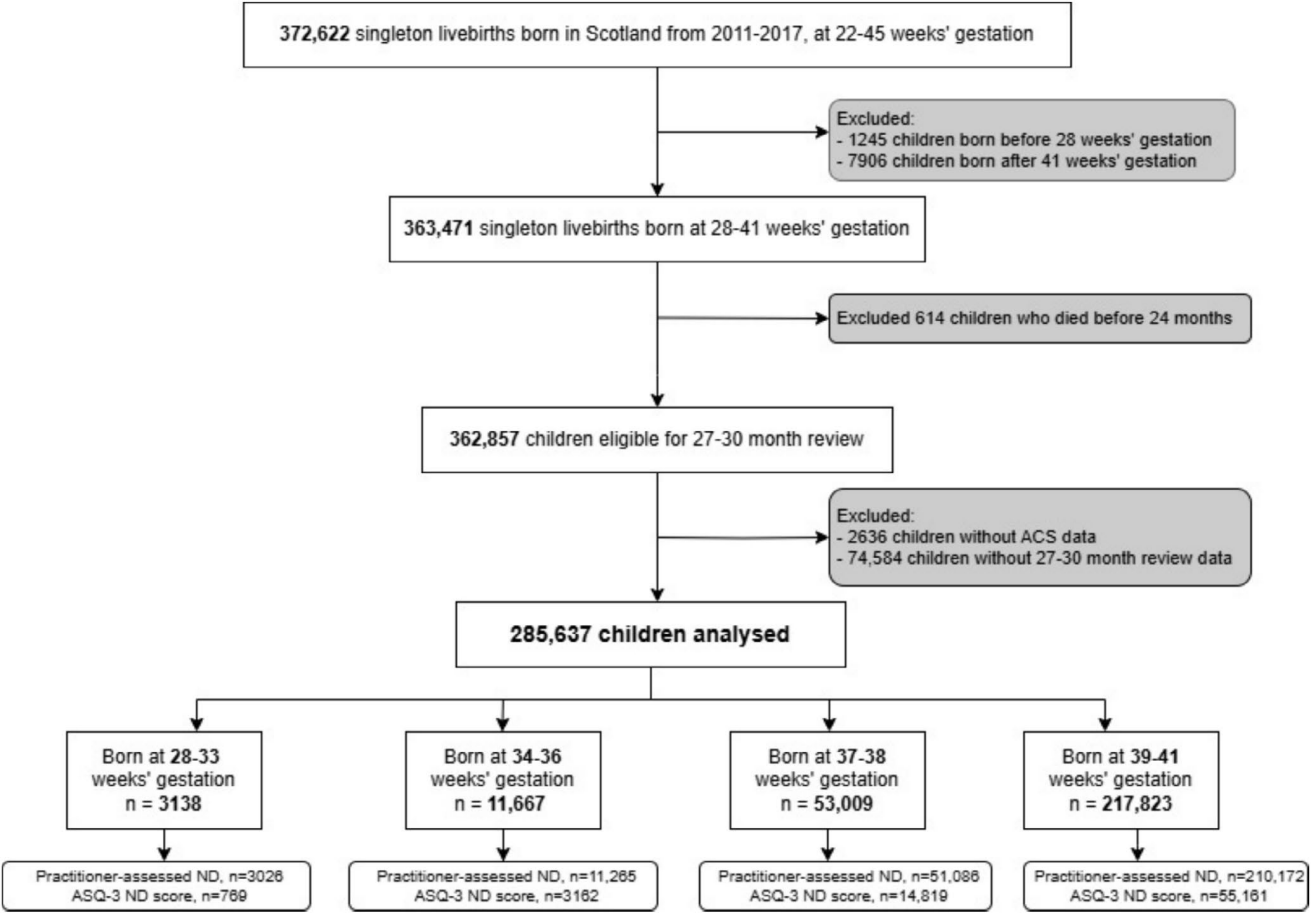
Derivation of the study cohort. “Practitioner‐assessed ND” refers to children with complete outcome data on practitioner concern about neurodevelopment and “ASQ‐3 ND score” refers to children with complete data on ASQ‐3 neurodevelopment score. ACS, antenatal corticosteroids; ASQ‐3, Ages and Stages Questionnaire Third Edition; *n* = number of children; ND, neurodevelopment.

Figure 1 shows derivation of the study population. From SMR02, 363 471 singleton live born children born at 28–41 weeks' gestation were identified, of which 614 died before age 24 months. This left 362 987 children eligible for the 27–30 month review. Exclusion of mother–child dyads with missing data on ACS exposure (*n* = 2636) and 27–30 month reviews (*n* = 74 584) left 285 637 children eligible for the analysis (78.2% of children eligible for review), born to 232 589 mothers.

Attrition analyses comparing children included in the current study with children eligible for inclusion, who were excluded because of missing data, are shown in Table S4A. Of all children in the study, 275 549 (96.5%) had complete data on practitioner‐assessed developmental domains, from which the outcome practitioner concern about neurodevelopment was determined, and 74 032 (25.9%) had an ASQ‐3 score recorded for all ASQ‐3 domains, which enabled calculation of ASQ‐3 neurodevelopment scores. Attrition analyses in Table S4B,C described in Text S3, compare children with and without data on specific outcomes.

### 3.1 Demographics

In total, 10 196 (3.6%) of children in the cohort were exposed to ACS. Gestation‐specific ACS exposure rates were as follows: 2767 (88.2%) of 3138 children born at 28–33 weeks' gestation, 4570 (39.2%) of 11 667 children born at 34–36 weeks' gestation, 2268 (4.3%) of 53 009 children born at 37–38 weeks' gestation, and 591 (0.3%) of 217 823 children born at 39–41 weeks' gestation. Descriptive statistics of the study population according to ACS exposure, stratified by gestational age at birth, are presented in Tables 1 and 2. In ACS‐exposed children across all gestational age categories, mean birthweights were lower, compared to non‐ACS‐exposed children (Tables 1 and 2). Gestation‐specific associations of covariates with ACS exposure are described in Text S2. The mean child age at review was 28.8 (SD 1.6) months.

**TABLE 1:** Maternal characteristics of the study population, according to ACS exposure.

	Born at 28–33 weeks' gestation			Born at 34–36 weeks' gestation			Born at 37–38 weeks' gestation			Born at 39–41 weeks' gestation		
	All	Non‐ACS‐exposed	ACS‐ exposed	All	Non‐ACS‐exposed	ACS‐ exposed	All	Non‐ACS‐exposed	ACS‐exposed	All	Non‐ACS‐exposed	ACS‐exposed
	*N* = 3138	*N* = 371 (11.8%)	*N* = 2767 (88.2%)	*N* = 11 667	*N* = 7097 (60.8%)	*N* = 4570 (39.2%)	*N* = 53 009	*N* = 50 741 (95.7%)	*N* = 2268 (4.3%)	*N* = 217 823	*N* = 217 232 (99.7%)	*N* = 591 (0.3%)
**Maternal age at delivery (years)**, mean (SD)	29.1 (6.2)	28.8 (6.5)	29.2 (6.2)	29.6 (6.1)	29.5 (6.1)	29.7 (6.1)	29.7 (5.9)	29.7 (5.9)	30.8 (5.9)	29.4 (5.7)	29.4 (5.7)	28.5 (6.3)
Missing	0			0			0			0		
**Parity: Primiparous**, *N* (%)	1567 (50.3)	172 (46.5)	1395 (50.8)	5178 (44.6)	3121 (44.2)	2057 (45.2)	20 032 (37.9)	19 374 (38.3)	658 (29.1)	93 256 (43.0)	93 033 (43.0)	223 (37.8)
Missing	20	1	19	48	30	18	178	172	6	894	893	1
**Maternal Body Mass Index** ^1^												
Underweight (≤ 18.5 kg/m^2^)	142 (5.0)	24 (7.0)	118 (4.7)	446 (4.1)	281 (4.2)	165 (3.9)	1725 (3.5)	1667 (3.5)	58 (2.7)	5177 (2.5)	5157 (2.5)	20 (3.7)
Healthy weight (18.5–24.9 kg/m^2^)	1295 (45.7)	166 (48.7)	1129 (45.3)	4954 (45.9)	3164 (47.9)	1790 (42.8)	21 918 (44.3)	21 121 (44.6)	797 (36.9)	98 692 (48.3)	98 453 (48.4)	239 (44.4)
Overweight (25–29.9 kg/m^2^)	758 (26.8)	91 (26.7)	667 (26.8)	2877 (26.7)	1699 (25.7)	1178 (28.2)	13 295 (26.9)	12 712 (26.8)	583 (27.0)	57 580 (28.2)	57 428 (28.2)	152 (28.3)
Obese (≥ 30 kg/m^2^)	636 (22.5)	60 (17.6)	576 (23.1)	2516 (23.3)	1468 (22.2)	1048 (25.1)	12 576 (25.4)	11 856 (25.0)	720 (33.4)	42 683 (20.9)	42 556 (20.9)	127 (23.6)
Missing	307	30	277	874	485	389	3495	3385	110	13 691	13 638	53
**Maternal diabetes** ^2^, *N* (%)	192 (6.3)	18 (5.0)	174 (6.4)	1008 (8.8)	516 (7.4)	492 (11.0)	4091 (7.8)	3804 (7.6)	287 (12.8)	3367 (1.6)	3349 (1.6)	18 (3.1)
Missing	79	13	66	169	86	83	840	812	28	3641	3634	7
**Smoking status** ^1^, *N* (%)	800 (27.4)	133 (38.0)	667 (25.9)	2739 (24.5)	1661 (24.4)	1078 (24.7)	10 519 (20.6)	10 061 (20.6)	458 (21.0)	34 656 (16.4)	34 515 (16.4)	141 (24.7)
Missing	217	21	196	478	276	202	1906	1816	90	6791	6770	21
**Neighbourhood deprivation (SIMD deciles)**, mean (SD)	4.6 (2.8)	4.2 (2.7)	4.6 (2.9)	4.7 (2.9)	4.8 (2.9)	4.7 (2.9)	4.8 (2.9)	4.8 (2.9)	5.0 (3.0)	5.1 (2.9)	5.1 (2.9)	4.7 (2.9)
Missing	2	0	2	12	11	1	58	56	2	260	260	0

*Note:* Percentages are proportions of non‐ACS‐exposed or ACS‐exposed children with data available, in each gestational age category, and may not total 100% in view of rounding.

Abbreviations: ACS, antenatal corticosteroids; *N*, number of children; SD, standard deviation; SIMD, Scottish Index of Multiple Deprivation (1 = most deprived decile, 10 = least deprived decile).

^1^ Assessed at first antenatal appointment.

^2^ Any maternal diabetes – includes pre‐existing and gestational diabetes.

**TABLE 2:** Child characteristics and outcomes of the study population, according to ACS exposure.

	Born at 28–33 weeks' gestation			Born at 34–36 weeks' gestation			Born at 37–38 weeks' gestation			Born at 39–41 weeks' gestation		
	All	Non‐ACS‐exposed	ACS‐ exposed	All	Non‐ACS‐exposed	ACS‐ exposed	All	Non‐ACS‐exposed	ACS‐exposed	All	Non‐ACS‐exposed	ACS‐exposed
	*N* = 3138	*N* = 371 (11.8%)	*N* = 2767 (88.2%)	*N* = 11 667	*N* = 7097 (60.8%)	*N* = 4570 (39.2%)	*N* = 53 009	*N* = 50 741 (95.7%)	*N* = 2268 (4.3%)	*N* = 217 823	*N* = 217 232 (99.7%)	*N* = 591 (0.3%)
**Child characteristics**												
**Child sex** **irl**, *N* (%)	1348 (43.0)	164 (44.2)	1184 (42.8)	5374 (46.1)	3242 (45.7)	2132 (46.7)	25 113 (47.4)	24 005 (47.3)	1108 (48.9)	106 958 (49.1)	106 673 (49.1)	285 (48.2)
Missing	0			0			0			0		
**Year of birth**, *N* (%)												
2011	485 (15.5)	57 (15.4)	428 (15.5)	1651 (14.2)	1087 (15.3)	564 (12.3)	7342 (13.9)	7227 (14.2)	115 (5.1)	34 311 (15.8)	34 249 (15.8)	62 (10.5)
2012	437 (13.9)	52 (14.0)	385 (13.9)	1718 (14.7)	1105 (15.6)	613 (13.4)	7462 (14.1)	7280 (14.3)	182 (8.0)	34 135 (15.7)	34 055 (15.7)	80 (13.5)
2013	516 (16.4)	66 (17.8)	450 (16.3)	1650 (14.1)	994 (14.0)	656 (14.4)	7501 (14.2)	7190 (14.2)	311 (13.7)	33 371 (15.3)	33 266 (15.3)	105 (17.8)
2014	497 (15.8)	68 (18.3)	429 (15.5)	1762 (15.1)	1033 (14.6)	729 (16.0)	8103 (15.3)	7648 (15.1)	455 (20.1)	33 942 (15.6)	33 837 (15.6)	105 (17.8)
2015	508 (16.2)	60 (16.2)	448 (16.2)	1970 (16.9)	1157 (16.3)	813 (17.8)	8482 (16.0)	8056 (15.9)	426 (18.8)	32 289 (14.8)	32 181 (14.8)	108 (18.3)
2016	420 (13.4)	45 (12.1)	375 (13.6)	1758 (15.1)	1069 (15.1)	689 (15.1)	8596 (16.2)	8156 (16.1)	440 (19.4)	30 824 (14.2)	30 738 (14.1)	86 (14.6)
2017	275 (8.8)	23 (6.2)	252 (9.1)	1158 (9.9)	652 (9.2)	506 (11.1)	5523 (10.4)	5184 (10.2)	339 (14.9)	18 951 (8.7)	18 906 (8.7)	45 (7.6)
Missing	0			0			0			0		
**Gestational age at birth**, mean (SD)	31.4 (1.6)	31.4 (1.6)	31.4 (1.6)	35.5 (0.7)	35.8 (0.5)	35.0 (0.8)	37.7 (0.5)	37.7 (0.5)	37.5 (0.5)	39.9 (0.8)	39.9 (0.8)	39.5 (0.7)
**Birthweight** **(g)**, mean (SD)	1717.1 (466.0)	1789.5 (549.7)	1707.4 (452.9)	2640.1 (495.8)	2723.8 (465.5)	2509.6 (513.1)	3162.8 (481.6)	3165.1 (478.5)	3111.2 (543.5)	3560.7 (455.1)	3561.2 (454.9)	3388.9 (490.0)
Missing	23	5	18	33	10	23	74	69	5	320	317	3
**Child age at time of review (months),** mean (SD)	28.8 (1.7)	28.5 (1.6)	28.8 (1.7)	28.8 (1.6)	28.7 (1.6)	28.9 (1.6)	28.9 (1.6)	28.9 (1.6)	28.7 (1.6)	28.8 (1.6)	28.8 (1.6)	28.7 (1.7)
**Child outcomes**												
**Practitioner concerns about neurodevelopment** *N* (%)	977 (32.3)	137 (38.8)	840 (31.4)	2646 (23.5)	1562 (22.8)	1084 (24.6)	10 722 (21.0)	10 290 (21.0)	432 (19.7)	35 429 (16.9)	35 313 (16.8)	116 (20.2)
Missing	112	18	94	402	234	168	1923	1848	75	7651	7634	17
**ASQ‐3 neurodevelopment score**, mean (SD)	47.9 (9.4)	46.9 (10.5)	48.0 (9.2)	50.0 (8.6)	50.0 (8.6)	50.1 (8.7)	50.9 (8.5)	50.9 (8.5)	50.7 (8.4)	52.0 (7.5)	52.0 (7.5)	50.7 (9.0)
Missing	2368	293	2075	8498	5230	3268	38 163	36 722	1441	162 576	162 132	444

*Note:* Percentages are proportions of non‐ACS‐exposed or ACS‐exposed children with data available, in each gestational age category, and may not total 100% in view of rounding.

Abbreviations: ACS, antenatal corticosteroids; ASQ‐3, Ages & Stages Questionnaire Third Edition; *N*, number of children; SD, standard deviation.

Gestation‐specific associations of covariates with neurodevelopmental outcomes are presented in Table S5A,B and described in Text S2. Across all gestational age groups, multiparity, maternal obesity, antenatal smoking and neighbourhood deprivation were associated with increased odds of practitioner concerns about neurodevelopment, while older maternal age and female child sex were associated with reduced odds of neurodevelopmental concerns. In all gestational age categories, antenatal smoking was associated negatively with ASQ‐3 neurodevelopment scores, and girls and older children had higher ASQ‐3 neurodevelopment scores.

### 3.2 Children Born at 28–33 Weeks' Gestation

In children born at 28–33 weeks' gestation, practitioners recorded concerns about neurodevelopment in 31.4% (840/2673) of ACS‐exposed children and in 38.8% (137/353) of non‐ACS‐exposed children (Table 3). After adjustment for child age and sex (Model 2), ACS exposure was associated with reduced odds of practitioner concerns about neurodevelopment, and this association remained statistically significant after adjustment for maternal and perinatal covariates (Model 3). However, ACS exposure was not associated with ASQ‐3 neurodevelopment scores in unadjusted or adjusted analyses (Table 4).

**TABLE 3:** The association of ACS exposure with practitioner concerns about neurodevelopment, stratified by gestational age.

Gestational age at birth (weeks' gestation)	Presence of practitioner concerns about neurodevelopment								
	No. of children with concern/No. of children with outcome data (%)			Model 1^a^ Unadjusted		Model 2^b^ Minimally adjusted		Model 3^c^ Fully adjusted	
	Total	Non‐ACS‐exposed	ACS‐exposed	OR (95% CI)	*p*	OR (95% CI)	*p*	OR (95% CI)	*p*
28–33	977/3026 (32.3%)	137/353 (38.8%)	840/2673 (31.4%)	**0.72 (0.58; 0.91)**	**0.005**	**0.71 (0.57; 0.90)**	**0.004**	**0.79 (0.62; 0.999)**	**0.049**
**34–36**	2646/11 265 (23.5%)	1562/6863 (22.8%)	1084/4402 (24.6%)	**1.11 (1.02; 1.21)**	**0.02**	**1.11 (1.01; 1.21)**	**0.03**	**1.11 (1.01; 1.21)**	**0.03**
37–38	10 722/51 086 (21.0%)	10 290/48 893 (21.0%)	432/2193 (19.7%)	0.92 (0.83; 1.03)	0.13	0.94 (0.84; 1.04)	0.23	0.96 (0.85; 1.07)	0.42
39–41	35 429/210 172 (16.9%)	35 313/209 598 (16.8%)	116/574 (20.2%)	**1.25 (1.02; 1.53)**	**0.03**	**1.25 (1.02; 1.54)**	**0.03**	1.11 (0.90; 1.37)	0.33

*Note:* Bold values indicates statistically significant.

Abbreviations: ACS, antenatal corticosteroids; CI, confidence intervals; *N*, number; OR, odds ratio.

^a^ Model 1 is unadjusted.

^b^ Model 2 adjusts for child sex and child age at review.

^c^ Model 3 adjusts for child sex, child age at review and maternal age, maternal BMI, smoking status at first antenatal appointment, parity, maternal diabetes, year of birth and neighbourhood deprivation.

**TABLE 4:** The association of ACS exposure with ASQ‐3 neurodevelopment score, stratified by gestational age.

Gestational age at birth (weeks' gestation)	ASQ‐3 neurodevelopment score								
	Number of children with ASQ‐3 neurodevelopment score			Model 1^a^ Unadjusted		Model 2^b^ Minimally adjusted		Model 3^c^ Fully adjusted	
	Total	Non‐ACS‐exposed	ACS‐exposed	B (95% CI)	*p*	B (95% CI)	*p*	B (95% CI)	*p*
28–33	769	78	691	0.06 (−0.18; 0.30)	0.62	0.04 (−0.20; 0.27)	0.76	−0.002 (−0.24; 0.24)	0.99
**34–36**	3162	1862	1300	0.04 (−0.03; 0.11)	0.27	0.02 (−0.05; 0.09)	0.58	0.03 (−0.04; 0.10)	0.45
37–38	14 819	13 994	825	−0.04 (−0.11; 0.04)	0.34	−0.03 (−0.10; 0.04)	0.47	−0.02 (−0.09; 0.05)	0.53
39–41	55 161	55 014	147	−0.09 (−0.25; 0.07)	0.25	−0.11 (−0.26; 0.05)	0.16	−0.08 (−0.23; 0.07)	0.31

*Note:* The ASQ‐3 neurodevelopment score is a sum‐score of ASQ‐3 scores recorded in all five developmental domains, including communication, gross motor, fine motor, problem solving, and personal/social. Only children with ASQ‐3 scores recorded in all five domains have been included in analyses.

Abbreviations: ACS, antenatal corticosteroids; ASQ‐3, Ages and Stages Questionnaire Third Edition; B, Unstandardised regression coefficient, with the outcome variable expressed in standard deviation units*; CI, confidence interval.

^a^ Model 1 is unadjusted.

^b^ Model 2 adjusts for child sex and child age at review.

^c^ Model 3 adjusts for child sex, child age at review and maternal age, maternal BMI, smoking status at first antenatal appointment, parity, maternal diabetes, year of birth and neighbourhood deprivation.

* The B values and 95% CIs for models 1–3 can be interpreted as reflecting the mean unadjusted and adjusted group differences of ACS‐exposed versus non‐ACS‐exposed children in the ASQ‐3 neurodevelopmental scores in standard deviation units, where the mean score in standard deviation units for whole cohort is 0 and standard deviation is 1.

### 3.3 Children Born at 34–36 Weeks' Gestation

In children born at 34–36 weeks' gestation, practitioners recorded concerns about neurodevelopment in 24.6% (1084/4402) of ACS‐exposed children and 22.8% (1562/6863) of non‐ACS‐exposed children (Table 3). After adjustment for age and sex (Model 2), ACS exposure was statistically significantly associated with increased odds of practitioner concerns about neurodevelopment, and this association persisted after adjustment for maternal and perinatal covariates (Model 3). No associations were found between ACS exposure and ASQ‐3 neurodevelopment scores in unadjusted or adjusted analyses (Table 4).

### 3.4 Children Born at 37–38 Weeks' Gestation

In children born at 37–38 weeks' gestation, practitioners recorded concerns about neurodevelopment in 19.7% (432/2193) of ACS‐exposed children and in 21.0% (102.90/48893) of non‐ACS‐exposed children (Table 3). In unadjusted and adjusted analyses, ACS exposure was not associated with practitioner concerns about neurodevelopment, or with ASQ‐3 neurodevelopment scores (Table 4).

### 3.5 Children Born at 39–41 weeks' Gestation

In children born at 39–41 weeks' gestation, practitioners recorded concerns about neurodevelopment in 20.2% (116/574) of ACS‐exposed children and in 16.8% (35 313/201598) of non‐ACS‐exposed children (Table 3). After adjustment for child age and sex (Model 2), ACS exposure was associated with a statistically significant increased odds of practitioner concerns about neurodevelopment. However, this association was partially explained by maternal and perinatal covariates and was no longer significant in Model 3. ACS exposure was not associated with ASQ‐3 neurodevelopment scores (Table 4).

Across all gestational age groups, ACS exposure did not interact with child sex in predicting practitioner concerns about neurodevelopment (all *p*‐values ≥ 0.10). There was an interaction of ACS exposure by child sex on ASQ‐3 neurodevelopment scores (*p* = 0.03 in Models 1–2; *p* = 0.04 in Model 3) in children born at 28–33 weeks' gestation. However, ACS exposure was not associated with ASQ‐3 neurodevelopment scores in either sex (all *p*‐values ≥ 0.07). There were no interactions of ACS by child sex on ASQ‐3 neurodevelopment scores in other gestational age groups (*p* ≥ 0.10).

### 3.6 Sensitivity Analyses

In sensitivity analyses restricted to each mother's first child born into the cohort, ACS exposure was still associated with statistically significant decreased odds of practitioner concerns about neurodevelopment in children born at 28–33 weeks' gestation. In children born at 34–36 weeks' gestation, ACS exposure was associated with increased odds of practitioner concerns about neurodevelopment, but this association was no longer statistically significant after adjustment for maternal and perinatal confounders (Table S6A). Among children born at 34–36 weeks' gestation, ACS exposure was also associated with higher ASQ‐3 neurodevelopment scores (Table S6B).

## 4 Discussion

### 4.1 Main Findings

This large population‐based cohort study of associations between ACS exposure and neurodevelopmental outcomes at 27–30 months of age found that in children born at 28–33 weeks' gestation, ACS exposure was associated with a statistically significantly reduced odds of practitioner concerns about their neurodevelopment. Children born at 34–36 weeks' gestation who were ACS‐exposed had statistically significantly increased odds of practitioner concerns about neurodevelopment than non‐ACS‐exposed children born at this gestation. In children born at 39–41 weeks' gestation, associations that were observed between ACS exposure and increased odds of practitioner neurodevelopmental concerns were attenuated to non‐significance after adjusting for maternal and perinatal confounders.

ACS exposure was not associated with practitioner‐identified neurodevelopmental concerns in children born at 37–38 weeks' gestation, nor with parent‐assessed continuous neurodevelopment in any gestational age group.

### 4.2 Interpretation

Gestational age at birth must be carefully considered in an evaluation of ACS with childhood neurodevelopment [16, 17, 18, 19]. Several studies which explored these associations adjusted for gestational age at birth in regression models [9, 12, 13, 14, 35], which risks introducing statistical multicollinearity and biasing effect size estimates, since the prevalence of ACS exposure markedly decreases with increasing gestational age. By stratifying analyses into four gestational age groups, our study avoided this multicollinearity bias, and offered new insight on the interplay between ACS exposure, gestational age at birth and child neurodevelopment.

Our findings reiterate the importance of targeting ACS use to those who will benefit most, while reducing potential harm from unnecessary treatments. The finding that ACS exposure in children born between 28‐33 weeks' gestation was associated with statistically significantly reduced neurodevelopmental concerns in early childhood is promising. This association was robust to sensitivity analysis, and this finding extends those of the meta‐analysis which concluded that in children born before 28 weeks' gestation, ACS exposure was associated with reduced risk of neurodevelopmental impairment at 18–22 months [6].

However, the present findings in children born ≥ 37 weeks' gestation partly contrast with observational studies from Finland [12, 14] and Canada [13], which demonstrated that ACS exposure in term‐born children was associated with an increased risk of mental and behavioural disorders [12], specific developmental disorders [14], and suspected or proven neurodevelopmental problems [13], independently of covariates. Although the present study found an association between ACS exposure and increased odds of practitioner concerns about neurodevelopment in children born at 39–41 weeks' gestation, this diminished after adjusting for maternal and perinatal confounders. Furthermore, no association was found between ACS exposure and continuously assessed neurodevelopment in children born at 39–41 weeks' gestation, nor with either neurodevelopment index in children born at 37–38 weeks' gestation. Stratification of term‐born children into these specific gestational age groups is justified since neurocognitive development of term‐born children improves with each week of gestation at birth [40]. In the present study, the sample size of term‐born ACS‐exposed children, and their duration of follow‐up, were smaller and shorter, respectively, compared to the previous studies [12, 13, 14], which may account for differences in findings.

In children born at 34–36 weeks' gestation, the present findings of statistically significantly increased neurodevelopmental concerns following ACS exposure concur with a Canadian cohort study, which reported an association between ACS exposure and increased investigation of suspected neurodevelopmental problems in children born late preterm [9]. In sensitivity analyses among firstborn children of mothers in the cohort, born at 34–36 weeks' gestation, ACS exposure was unexpectedly associated with higher continuous neurodevelopment scores. However, this finding must be interpreted with caution, as it was not present in the whole cohort, and ACS exposure was also associated with increased odds of practitioner‐identified neurodevelopmental concerns in this subsample.

It is of note, however, that the associations we observed between ACS exposure and reduced odds of neurodevelopmental concerns in children born at 28–33 weeks' gestation, and between ACS exposure and increased odds of neurodevelopmental concerns in children born at 34–36 weeks' gestation, although statistically significant, were both of small effect size. After adjusting for all covariates, in children born at 28–33 weeks' gestation, those who were not exposed to ACS had a 27% increased odds of neurodevelopmental concerns, while in children born at 34–36 weeks' gestation, those who were exposed to ACS had an 11% increased odds of neurodevelopmental concerns. Both of these effect sizes are small, according to Cohen's effect size classification [42]. Our findings may illustrate that small effects of limited clinical relevance can potentially become statistically significant in sufficiently large study samples. Indeed, all statistical hypothesis testing, whether in small or large study samples, include the possibility of false positive and false negative findings, and our findings must be interpreted with caution. However, our findings are consistent with evidence from previous animal and epidemiological studies on the effects of ACS exposure [6, 9, 43]. Furthermore, it is known from epidemiological studies that the aetiology of neurodevelopmental concerns is multifactorial, with individual risk and protective factors exerting effects of only small sizes [44, 45, 46].

To our knowledge, only one cohort study has previously examined associations of ACS exposure with individual variations in neurodevelopment using validated questionnaires [35], and it reported an association of ACS with a higher prevalence of mother‐assessed failure to meet age‐appropriate developmental milestones in communication, problem‐solving and personal social skills in children born preterm and at term [35]. While that study used ASQ‐3 domain score thresholds to indicate failure to meet domain‐specific developmental milestones [35], the present study examined the association of ACS with a continuous ASQ‐3 neurodevelopment score, combining data across multiple developmental domains. Our more precise stratification of children based on gestational age at birth found no consistent associations of ACS exposure with continuously assessed child neurodevelopment.

### 4.3 Strengths and Limitations

Key strengths of this study include its longitudinal design and large sample size. This enabled analyses to be conducted within four gestational age groups, which provided important insights into the associations of ACS exposure with childhood neurodevelopment. This population‐wide study had good representativeness [24, 25], and through record linkage with nationwide child health reviews, minimised the selection bias inherent to longitudinal data collected by questionnaires or from referral to medical settings. This enabled evaluation of associations between ACS exposure and the full spectrum of variation in neurodevelopment, including both continuous and categorical indices, adding depth and validity to the findings.

The study limitations include the lack of information on gestational age at ACS administration, corticosteroid formulation and dosage, indications for ACS, underlying causes/mechanisms of preterm birth such as infection, or exposure to antibiotics. This allows potential for residual confounding. Dose‐dependent effects of ACS exposure on neurodevelopment could not be assessed, which further limits causal inference. Furthermore, the potential mediating, moderating and/or confounding effects of fetal growth restriction, congenital anomalies, genetic disorders, and maternal hypertensive disorders were not assessed. Missing data on ACS exposure and 27–30 month reviews led to exclusion of some children from the study, and the representativeness of the study population may be limited by social inequalities in health care. Children from the most deprived neighbourhoods are less likely to attend health reviews [47], and children excluded from our study had higher rates of neighbourhood deprivation. Numbers of treatment discordant siblings in each subsample were inadequate to undertake sibpair analyses, thus genetic or shared familial confounding may have contributed to our findings.

Although child follow‐up took place at 27–30 months of age, not all neurodevelopmental problems will have emerged by then [48]. Additionally, the identification of a “practitioner concern” is not commensurate with a confirmed neurodevelopmental problem. Furthermore, the continuous ASQ‐3 neurodevelopmental outcome data were limited to a subgroup of children, comprising only 25.9% of the original study cohort. It became national policy in Scotland to use the ASQ‐3 questionnaire at the 27–30 month review in 2017 [20] but its implementation has been variable [25]; therefore, ASQ‐3 data were limited to children born in 2015–2017, of whom 66.7% had complete data.

## 5 Conclusion

This population‐based cohort study of ACS exposure and neurodevelopment in early childhood showed that after adjusting for child age, sex, maternal and perinatal covariates, statistically significant associations were observed between ACS exposure and reduced odds of practitioner‐identified neurodevelopmental concerns in children born at 28–33 weeks' gestation, and with increased odds of practitioner‐identified neurodevelopmental concerns in children born at 34–36 weeks' gestation. Associations between ACS exposure and increased odds of practitioner‐identified neurodevelopmental concerns in children born at 39–41 weeks' gestation were partly attenuated after adjustment for maternal and perinatal covariates. However, the effect sizes of all the aforementioned associations were small. No consistent associations between ACS exposure and continuously assessed neurodevelopment were observed. Future studies should include school performance and educational achievement outcomes to assess potential associations of ACS with neurodevelopment beyond early childhood.

## Author Contributions

E.M.F., M.L‐.P., C.L., F.D., H.Z., K.A., J.V.B., D.B., K.E., L.F., A.F., M.G., C.G‐.B., L.H.P., J.E.M., B.W.M., S.R.M., J.N., D.R., E.S., A.S., J.P.V., R.W., B.J., E.K., R.R. and S.J.S. contributed to the study concept and design. Data analysis was conducted by E.M.F. and M.L‐.P. with contributions from C.L., F.D., R.M.R. and S.J.S. E.M.F. and M.L‐.P. wrote the original draft, and F.D., H.Z., K.A., J.V.B., D.B., K.D., K.E., A.F., M.G., C.G‐.B., L.H.P., J.E.M., B.W.M., S.R.M., E.S., T.S., A.S., J.P.V., R.W., E.Mc.G., B.J., R.M.R. and S.J.S. reviewed and edited the paper. E.M.F. and M.L‐.P. coordinated the study. R.M.R. and S.J.S. supervised the study. Funding was obtained by S.J.S.

## Ethics Statement

The Co‐OPT ACS study received ethical approval from the Academic and Clinical Centre Office for Research and Development Medical Research Ethics Committee (19‐HV‐063) on 9th December 2019, and information governance approval from the Public Benefit and Privacy Panel for Health and Social Care (1718–0054) on 16th October 2019.

## Conflicts of Interest

The Co‐OPT ACS study is funded through a Wellcome Trust Clinical Career Development Fellowship grant (Funding Reference number 209560/Z/17) awarded to SJS. ML‐P is supported by grants from the Academy of Finland (330206). FD is supported by the Swiss National Science Foundation (P500PM_206634) and the Bangeter‐Rhyner Foundation. HZ is supported by a UNSW Scientia Program Award. DB is supported by a National Health and Medical Research Council Investigator Grant (GTN1175744). Research at Murdoch Children's Research Institute (DB, JEM) is supported by the Victorian Government's Operational Infrastructure Program. KD is funded by Health Education England (HEE)/NIHR. CG‐B is supported by research grants from NHLBI, NICHD and NIHMD, and has received research funding from HealthCore Inc/SERA Prognostics Inc. and MIRVIE Inc. LHP is supported by a Borregaard Clinical Ascending Investigator Grant from the Novo Nordisk Foundation (NNF18OC0054457). JPV is supported by an Australian National Health and Medical Research Council (NHMRC) Investigator grant (GNT1194248). EMcG acknowledges support of the NIHR North West Coast Scholars' Programme. RMR acknowledges the support of the British Heart Foundation (RE/18/5/34216). The remaining authors declare no conflicts of interest.

## Supporting information


Figure S1.

Tables S1‐S6.

Text S1‐S3.

## Data Availability

Patient‐level data in this study cannot be shared publicly because of data protection and confidentiality requirements established by Public Health Scotland and the National Records of Scotland (the data holders), as the dataset contains sensitive and potentially identifying data. Data may be made available to approved researchers after securing relevant permissions from the data holders. Enquiries should be directed to the electronic Data Research and Innovation Service at Public Health Scotland (phs.edris@phs.scot).
