# Supplementary material for: Associations of Antenatal Corticosteroids With Neurodevelopment in Children Aged 27–30 Months: A Population‐Based Cohort Study
Source: BJOG. 2025 Feb 19;132(7):902–15. doi: 10.1111/1471-0528.18101 (PMC12051224; doi:10.1111/1471-0528.18101)
Supplement: Supplementary file 1 — Figure S1. [file BJO-132-902-s002.docx]

# Supporting information (Figure)

**
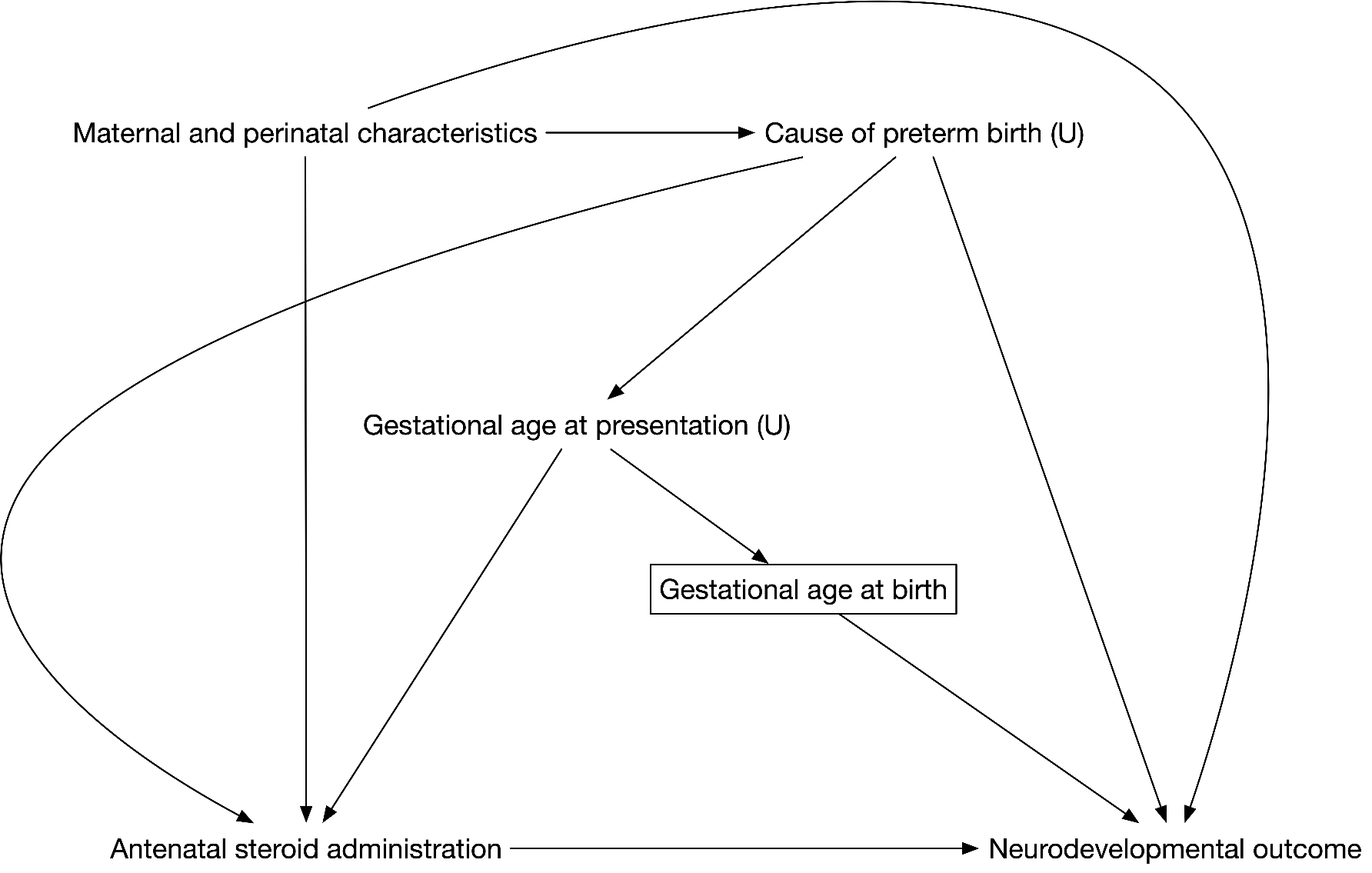
Figure S1. Directed acyclic graph of confounders of the association between antenatal corticosteroid exposure and neurodevelopmental outcome.**

U = Unknown.
