# Supplementary material for: Associations of Antenatal Corticosteroids With Neurodevelopment in Children Aged 27–30 Months: A Population‐Based Cohort Study
Source: BJOG. 2025 Feb 19;132(7):902–15. doi: 10.1111/1471-0528.18101 (PMC12051224; doi:10.1111/1471-0528.18101)
Supplement: Supplementary file 2 — Tables S1‐S6. [file BJO-132-902-s001.docx]

# Supporting information (Tables)

Table S1. Overview of key recommendations on use of antenatal corticosteroids in Scotland between 2010-2017.

Guidance has been considered from 2010-2017 as births are included from January 2011 until December 2017, and guidance shown applies to singleton pregnancies. Recommendations for the use of ACS in the presence of other comorbidities/complications (such as maternal diabetes) varies and can be found in original guidelines.

| **Primary source of guidelines** | **Recommended ACS dosing regimen** | **Standard practice** | **Special considerations** | | | |
| --- | --- | --- | --- | --- | --- | --- |
|  |  | **Administration at 24+0 - 33+6 weeks’ gestation** | **Administration before 24+0 weeks’ gestation** | **Administration at 34+0 - 36+6 weeks’ gestation** | **Administration before planned Caesarean section at 37+0 - 38+6 weeks’ gestation** | **Use of multiple doses of ACS (rescue or repeat doses)** |
| Royal College of Obstetricians & Gynaecologists (RCOG) ^1^  National Institute for Health and Care Excellence (NICE) ^2^ | Two doses 12mg intramuscular betamethasone, 24 hours apart  or  Four doses 6mg intramuscular dexamethasone, 12 hours apart | **√** - routine administration recommended^a^ | **NICE**, from 2015:  **(√)** – **23+0 – 23+6 weeks’ gestation**: discuss with the woman in context of her individual circumstances | **NICE**, from 2015:  **(√)** – **34+0 – 35+6 weeks’ gestation**: consider^a^  **RCOG**, from 2010 (archived 2016):  **√** - **34+0 - 34+6 weeks’ gestation**: routine administration recommended | **RCOG**, **from 2010 (archived 2016):**  **√** - recommended for all women with planned Caesarean **before 38+6 weeks’ gestation** | **NICE**, from 2015:  **X** – routine repeat courses not recommended |

^a^ if high risk of imminent medically-indicated or spontaneous preterm birth within the subsequent 7 days

**√** = routine use of ACS recommended. **(√)** = consider use of ACS. **X** = routine use of ACS not recommended.

Table S2. Outcome definitions, assessors and data rules.

| **Outcome & Assessor** | **Definition** | **Outcome type** | **Data rules & rationale** |
| --- | --- | --- | --- |
| **Practitioner concern about neurodevelopment**  Assessor: Practitioner | Presence of a concern recorded in at least one developmental domain assessed (review-dependent; domains shown in **Table S3**).  Both "newly suspected” concerns and “previously identified” concerns were equated to “Any concern recorded”. This outcome was used to index a problem in early child neurodevelopment. | Binary:   - Any concern recorded (coded as “Concern newly suspected” or “Concern previously identified”) - No concern recorded (coded as “No concerns”) | - If at least one developmental domain had missing data:   - Concern identified in at least one domain - Outcome = “Any concern recorded”   - No concerns identified any other domains - Outcome ≠ “No concerns”, and child excluded from analysis - The “problem solving” domain was introduced to the 27-30 month review in 2017 and it could not be mapped to any domains in reviews pre-2017 (**Table S3**), preventing comparison of outcomes from 27-30 month reviews which were undertaken pre- and post-2017. The problem solving domain was therefore excluded from this outcome^a^. - Some 27-30 month reviews included two sets of assessments for each developmental domain, based on “pre-2017” domains and “post-2017” domains. In these reviews, domains were mapped as per **Table S3**. A concern recorded either “pre-2017” or “post-2017” domain assessments was coded as “Any concern recorded”. |
| **ASQ-3**  **neurodevelopment score**  Assessor: Parent | Mean ASQ-3 score, calculated by the average of the five ASQ-3 domain scores^b^, each of which ranges from 0 to 60. The overall score served as an index of global neurodevelopment^c^. | Continuous score  (0 to 60) | - ASQ-3 scores were only calculated for children with ASQ-3 scores recorded for all five developmental domains. If at least one of five ASQ-3 domains had missing data, an ASQ-3 score was not determined, and the child was excluded from this analysis. |

ASQ-3 = Ages & Stages Questionnaire Third Edition^3^.

^a^ Assessments of development in problem solving domain were available for children who underwent 27-30 month reviews after 1^st^ April 2017 (see **Table S3**).

^b^ Since April 2017, health visitors have recorded individual ASQ-3 scores for five specific developmental domains in the questionnaire (communication, gross motor, fine motor, problem solving, and personal/social) ^4^. Each domain includes 6 questions, which are coded with three answer options: developmental skill is not yet present (0 points), the child can sometimes complete a task (5 points), or the child has already mastered a task (10 points)^3^.

^c^ Equivalent “ASQ-3 neurodevelopment scores” have been used in previous population studies to index global neurodevelopment ^5^

Table S3. Developmental domains assessed by practitioners at 27-30 month review, mapped before and after 1st April 2017 (adapted from *Early Child Development Statistics 2021/22, Public Health Scotland*^4^).

Before 1^st^ April 2017 (“Pre-2017”), the 27-30 month review assessed 9 domains, after which time (“Post-2017”) this was reduced to 8 domains^4^. Assessment of problem solving was introduced to the 27-30 month review on 1^st^ April 2017. The domains “Emotional development”, “Behavioural development” and “Attention” from reviews pre-2017 were mapped with the single domain “Emotional / Behavioural development” in reviews post-2017. The domain “Social development” pre-2017 was mapped with the “Personal / Social development” domain in reviews post-2017.

| **27-30 month review** | |
| --- | --- |
| **Pre-2017** | **Post-2017** |
| Speech, Language & Communication | Speech, Language & Communication |
| *-* | Problem Solving |
| Emotional development | Emotional / Behavioural development |
| Behavioural development |  |
| Attention |  |
| Gross Motor development | Gross Motor development |
| Fine Motor development | Fine Motor development |
| Social development | Personal / Social development |
| Vision | Vision |
| Hearing | Hearing |

Table S4(A-C). Attrition analyses of children excluded from the study.

Table S4-A. Attrition analyses of all children excluded from the study due to missing data on antenatal corticosteroid exposure or review data

|  | **Born at 28-33 weeks’ gestation** | | | **Born at 34-36 weeks’ gestation** | | | **Born at 37-38 weeks’ gestation** | | | **Born at 39-41 weeks’ gestation** | | |
| --- | --- | --- | --- | --- | --- | --- | --- | --- | --- | --- | --- | --- |
|  | **Excluded**^a^ | **Included** | **p value** | **Excluded**^a^ | **Included** | **p value** | **Excluded**^a^ | **Included** | **p value** | **Excluded**^a^ | **Included** | **p value** |
| **Number of children** (%) | 2109 (34.7) | 3138 (51.7) |  | 4927 (25.8) | 11,667 (61.0) |  | 14,808 (18.8) | 53,009 (67.1) |  | 55,376 (18.0) | 217,823 (70.8) |  |
| **Number of children exposed to ACS** (%)^b^ | 960 / 1296 (74.1) | 2767 / 3138 (88.2) | **<0.001** | 1256 / 3331 (37.7) | 4570 / 11667 (39.2) | 0.13 | 696 / 14733 (4.7) | 2268 / 53009 (4.3) | **0.02** | 135 / 55224 (0.3) | 591 / 217823 (0.3) | 0.72 |
| **Maternal Characteristics** | |  |  |  |  |  |  |  |  |  |  |  |
| **Maternal age at delivery (years)**, mean (SD) | 29.2 (6.4) | 29.1 (6.2) | 0.63 | 30.0 (6.0) | 29.7 (5.9) | 0.11 | 29.6 (6.0) | 29.7 (5.9) | **0.03** | 29.9 (5.6) | 29.9 (5.5) | 0.81 |
| **Parity: Primiparous**, N (%) | 941 / 2092 (45.0) | 1567 / 3118 (50.3) | **<0.001** | 2009 / 4894 (41.1) | 5178 / 11,619 (44.6) | **<0.001** | 5185 / 14,724 (35.2) | 20,032 / 52,831 (37.9) | **<0.001** | 22,139 / 55,086 (40.2) | 93,256 / 216,929 (43.0) | **<0.001** |
| **Maternal Body Mass Index (kg/m^2^)**, N (%) |  |  | 0.16 |  |  | 0.47 |  |  | 0.25 |  |  | **<0.001** |
| Underweight (<18.5) | 75 (4.1) | 142 (5.0) |  | 199 (4.5) | 446 (4.1) |  | 502 (3.6) | 1725 (3.5) |  | 1379 (2.7) | 5177 (2.5) |  |
| Normal weight (18.5-24.9) | 826 (45.7) | 1295 (45.7) |  | 2068 (46.5) | 4954 (45.9) |  | 6165 (44.7) | 21918 (44.3) |  | 25438 (49.3) | 98692 (48.3) |  |
| Overweight (25-29.9) | 459 (25.4) | 758 (26.8) |  | 1138 (25.6) | 2877 (26.7) |  | 3727 (27.0) | 13295 (26.9) |  | 14362 (27.8) | 57580 (28.2) |  |
| Obese (≥30) | 449 (24.8) | 636 (22.5) |  | 1045 (23.5) | 2516 (23.3) |  | 3391 (24.6) | 12576 (25.4) |  | 10407 (20.2) | 42683 (20.9) |  |
| **Maternal diabetes (any)**, N (%) | 116 / 2008 (5.8) | 192 / 3059 (6.3) | 0.47 | 390 / 4767 (8.2) | 1008 / 11498 (8.8) | 0.23 | 1113 / 14525 (7.7) | 4091 / 52169 (7.8) | 0.48 | 940 / 54273 (1.7) | 3367 / 214182 (1.6) | **0.01** |
| **Smoking at first antenatal appointment**, N (%) | 559 / 1908 (29.3) | 800 / 2921 (27.4) | 0.15 | 1183 / 4661 (25.4) | 2739 / 11189 (24.5) | 0.23 | 3003 / 14226 (21.1) | 10519 / 51103 (20.6) | 0.17 | 8768 / 53626 (16.4) | 34656 / 211032 (16.4) | 0.69 |
| **Child Characteristics** |  |  |  |  |  |  |  |  |  |  |  |  |
| **Child sex: Girl**, N (%) | 343 / 770 (44.5) | 1348 / 3138 (43.0) | 0.43 | 789 / 1731 (45.6) | 5374 / 11667 (46.1) | 0.71 | 941 / 1977 (47.6) | 25113 / 53009 (47.4) | 0.85 | 3966 / 8231 (48.2) | 105958 / 217823 (49.1) | 0.10 |
| **Year of birth**, N(%) |  |  | **<0.001** |  |  |  |  |  | **<0.001** |  |  | **<0.001** |
| 2011 | 318 (15.1) | 485 (15.5) |  | 609 (12.4) | 1651 (14.2) | **<0.001** | 1716 (11.6) | 7342 (13.9) |  | 7720 (13.9) | 34311 (15.8) |  |
| 2012 | 307 (14.6) | 437 (13.9) |  | 603 (12.2) | 1718 (14.7) |  | 1637 (11.1) | 7462 (14.1) |  | 7278 (13.1) | 34135 (15.7) |  |
| 2013 | 269 (12.8) | 516 (16.4) |  | 584 (11.9) | 1650 (14.1) |  | 1531 (10.3) | 7501 (14.2) |  | 6561 (11.8) | 33371 (15.3) |  |
| 2014 | 240 (11.4) | 497 (15.8) |  | 541 (11.0) | 1762 (15.1) |  | 1451 (9.8) | 8103 (15.3) |  | 6093 (11.0) | 33942 (15.6) |  |
| 2015 | 239 (11.3) | 508 (16.2) |  | 573 (11.6) | 1970 (16.9) |  | 1544 (10.4) | 8482 (16.0) |  | 5624 (10.2) | 32289 (14.8) |  |
| 2016 | 305 (14.5) | 420 (13.4) |  | 688 (14.0) | 1758 (15.1) |  | 1732 (11.7) | 8596 (16.2) |  | 5708 (10.3) | 30824 (14.2) |  |
| 2017 | 431 (20.4) | 275 (8.8) |  | 1329 (27.0) | 1158 (9.9) |  | 5197 (35.1) | 5523 (10.4) |  | 16392 (29.6) | 18951 (8.7) |  |
| **Gestational age at birth**, mean (SD) | 31.1 (1.7) | 31.4 (1.6) | **<0.001** | 35.3 (0.8) | 35.5 (0.7) | **<0.001** | 37.7 (0.5) | 37.7 (0.5) | 0.57 | 39.8 (0.8) | 39.9 (0.8) | **<0.001** |
| **Birthweight (g)**, mean (SD) | 1658.7 (507.8) | 1717.1 (466.0) | **<0.001** | 2612.7 (491.6) | 2641.6 (495.3) | **0.01** | 3154.1 (479.9) | 3162.8 (481.6) | 0.06 | 3548.0 (442.1) | 3576.9 (444.1) | **<0.001** |
| **Child age at time of review (months)**, mean (SD)^c^ | 29.2 (1.8) | 28.8 (1.7) | **<0.001** | 29.0 (1.8) | 28.8 (1.6) | **<0.001** | 28.6 (1.8) | 28.9 (1.6) | **<0.001** | 28.7 (1.8) | 28.8 (1.6) | **<0.001** |
| **Neighbourhood deprivation (SIMD deciles)**, mean (SD) | 4.5 (2.8) | 4.6 (2.8) | 0.20 | 4.6 (2.9) | 4.7 (2.9) | **0.01** | 4.9 (2.9) | 4.8 (2.9) | **0.02** | 5.0 (3.0) | 5.4 (2.8) | **<0.001** |
| **Child Outcomes**^d^ |  |  |  |  |  |  |  |  |  |  |  |  |
| **Practitioner concerns about neurodevelopment**^c^ | 185 / 572 (32.3) | 977 / 3026 (32.3) | 0.98 | 344 / 1240 (27.7) | 2646 / 11265 (23.5) | **<0.001** | 90 / 396 (22.7) | 10722 / 51086 (21.0) | 0.40 | 238 / 1451 (16.4) | 35429 / 210172 (16.9) | 0.65 |

Data are presented as numbers of children excluded or included with specific characteristic or outcome (% of children with available data for specific characteristic or outcome), or mean values (standard deviation).

ACS = Antenatal corticosteroids. ASQ-3 = Ages and Stages Questionnaire Third Edition. BMI = Body Mass Index. g = grams. N = Number of children. SD = Standard deviation. SIMD = Scottish Index of Multiple Deprivation (1=most deprived decile, 10=least deprived decile).

^a^ Children excluded from cohort due to missing ACS data or missing review data.

^b^ Children excluded from cohort due to missing review data.

^c^ Children excluded from cohort due to missing ACS data

^d^ The number of children with ASQ-3 data with missing ACS data was too small to allow comparison of ASQ scores between children excluded and included in the study based on availability of ACS data.

Table S4-B. Attrition analyses of children excluded from analyses of practitioner concerns about neurodevelopment

|  | **Born at 28-33 weeks’ gestation** | | | **Born at 34-36 weeks’ gestation** | | | **Born at 37-38 weeks’ gestation** | | | **Born at 39-41 weeks’ gestation** | | |
| --- | --- | --- | --- | --- | --- | --- | --- | --- | --- | --- | --- | --- |
|  | **Excluded** | **Included** | **p value** | **Excluded** | **Included** | **p value** | **Excluded** | **Included** | **p value** | **Excluded** | **Included** | **p value** |
| **Number of children** (%) | 2221 (42.3) | 3026 (57.7) |  | 5329 (32.1) | 11,265 (67.9) |  | 16,731 (24.7) | 51,086 (75.3) |  | 63,027 (23.1) | 210,172 (76.9) |  |
| **Number of children exposed to ACS** (%) | 1054 / 1408 (74.9) | 2673 / 3026 (88.3) | **<0.001** | 1424 / 3733 (38.1) | 4402 / 11,265 (39.1) | 0.31 | 771 / 16,656 (4.6) | 2193 / 51,086 (4.3) | 0.07 | 162 / 62,875 (0.3) | 574 / 210,172 (0.3) | 0.54 |
| **Maternal Characteristics** | |  |  |  |  |  |  |  |  |  |  |  |
| **Maternal age at delivery (years)**, mean (SD) | 29.2 (6.4) | 29.1 (6.2) | 0.48 | 29.4 (6.1) | 29.6 (6.1) | 0.19 | 29.6 (5.9) | 29.7 (5.9) | 0.05 | 29.36 (5.7) | 29.41 (5.7) | **0.02** |
| **Parity: Primiparous**, N (%) | 996 / 2203 (45.2) | 1512 / 3007 (50.3) | **<0.001** | 2185 / 5295 (41.3) | 5002 / 11,218 (44.6) | **<0.001** | 5953 / 16,645 (35.8) | 19,264 / 50,910 (37.8) | **<0.001** | 25,563 / 62,715 (40.8) | 89,832 / 209,300 (42.9) | **<0.001** |
| **Maternal Body Mass Index (kg/m^2^)**, N (%) |  |  | 0.11 |  |  | 0.59 |  |  | 0.25 |  |  | **<0.001** |
| Underweight (<18.5) | 78 (4.1) | 139 (5.1) |  | 208 (4.3) | 437 (4.2) |  | 561 (3.6) | 1666 (3.5) |  | 1576 (2.7) | 4980 (2.5) |  |
| Normal weight (18.5-24.9) | 869 (45.7) | 1252 (45.7) |  | 2227 (46.3) | 4795 (46.0) |  | 6940 (44.8) | 21,143 (44.2) |  | 28,686 (49.1) | 95,444 (48.4) |  |
| Overweight (25-29.9) | 483 (25.4) | 734 (26.8) |  | 1234 (25.6) | 2781 (26.7) |  | 4178 (27.0) | 12,844 (26.9) |  | 16,316 (27.9) | 55,626 (28.2) |  |
| Obese (≥30) | 472 (24.8) | 613 (22.4) |  | 1142 (23.7) | 2419 (23.2) |  | 3820 (24.6) | 12,147 (25.4) |  | 11,815 (20.2) | 41,275 (20.9) |  |
| **Maternal diabetes (any)**, N (%) | 123 / 2118 (5.8) | 185 / 2949 (6.3) | 0.51 | 417 / 5165 (8.1) | 981 / 11,100 (8.8) | 0.11 | 1272 / 16,410 (7.8) | 3932 / 50,284 (7.8) | 0.79 | 1047 / 61,740 (1.7) | 3260 / 206,715 (1.6) | **0.04** |
| **Smoking at first antenatal appointment**, N (%) | 595 / 2014 (29.5) | 764 / 2815 (27.1) | 0.07 | 1277 / 5047 (25.3) | 2645 / 10,803 (24.5) | 0.27 | 3347 / 16,062 (20.8) | 10,175 / 49267 (20.7) | 0.61 | 9921 / 60,961 (16.3) | 33503 / 203,697 (16.4) | 0.31 |
| **Child Characteristics** |  |  |  |  |  |  |  |  |  |  |  |  |
| **Child sex: Girl**, N (%) | 390 / 882 (44.2) | 1301 / 3026 (43.0) | 0.54 | 970 / 2133 (45.5) | 5193 / 11265 (46.1) | 0.60 | 1857 / 3900 (47.6) | 24197 / 51086 (47.4) | 0.77 | 7460 / 15882 (47.0) | 103464 / 210172 (49.2) | **<0.001** |
| **Year of birth**, N(%) |  |  | **<0.001** |  |  | **<0.001** |  |  | **<0.001** |  |  | **<0.001** |
| 2011 | 331 (14.9) | 472 (15.6) |  | 661 (12.4) | 1599 (14.2) |  | 1978 (11.8) | 7080 (13.9) |  | 8963 (14.2) | 33,068 (15.7) |  |
| 2012 | 322 (14.5) | 422 (13.9) |  | 659 (12.4) | 1662 (14.8) |  | 1868 (11.2) | 7231 (14.2) |  | 8352 (13.3) | 33,061 (15.7) |  |
| 2013 | 289 (13.0) | 496 (16.4) |  | 644 (12.1) | 1590 (14.1) |  | 1802 (10.8) | 7230 (14.2) |  | 7686 (12.2) | 32,246 (15.3) |  |
| 2014 | 251 (11.3) | 486 (16.1) |  | 585 (11.0) | 1718 (15.3) |  | 1713 (10.2) | 7841 (15.3) |  | 7124 (11.3) | 32,911 (15.7) |  |
| 2015 | 267 (12.0) | 480 (15.9) |  | 651 (12.2) | 1892 (11.4) |  | 1911 (11.4) | 8115 (15.9) |  | 6989 (11.1) | 30,924 (14.7) |  |
| 2016 | 319 (14.4) | 406 (13.4) |  | 763 (14.3) | 1683 (14.9) |  | 2088 (12.5) | 8240 (16.1) |  | 6942 (11.0) | 29,590 (14.1) |  |
| 2017 | 442 (19.9) | 264 (8.7) |  | 1366 (25.6) | 1121 (10.0) |  | 5371 (32.1) | 5349 (10.5) |  | 16,971 (26.9) | 18,372 (8.7) |  |
| **Gestational age at birth**, mean (SD) | 31.1 (1.7) | 31.4 (1.6) | **<0.001** | 35.3 (0.8) | 35.5 (0.8) | **<0.001** | 37.7 (0.5) | 37.7 (0.5) | 0.61 | 39.87 (0.8) | 39.92 (0.8) | **<0.001** |
| **Birthweight (g)**, mean (SD) | 1664.5 (510.2) | 1714.9 (462.8) | **<0.001** | 2573.2 (490.9) | 2640.9 (497.0) | **<0.001** | 3151.6 (479.8) | 3163.9 (481.7) | **<0.01** | 3545.3 (452.5) | 3561.4 (454.9) | **<0.001** |
| **Neighbourhood deprivation (SIMD deciles)**, mean (SD) | 4.4 (2.8) | 4.6 (2.9) | 0.07 | 4.6 (2.9) | 4.7 (2.9) | **0.01** | 4.8 (2.9) | 4.8 (2.9) | 0.21 | 5.2 (3.0) | 5.1 (2.9) | **<0.001** |

Table S4-C. Attrition analyses of children excluded from analyses of ASQ-3 neurodevelopment scores

|  | **Born at 28-33 weeks’ gestation** | | | **Born at 34-36 weeks’ gestation** | | | **Born at 37-38 weeks’ gestation** | | | **Born at 39-41 weeks’ gestation** | | |
| --- | --- | --- | --- | --- | --- | --- | --- | --- | --- | --- | --- | --- |
|  | **Excluded** | **Included** | **p value** | **Excluded** | **Included** | **p value** | **Excluded** | **Included** | **p value** | **Excluded** | **Included** | **p value** |
| **Number of children** (%) | 4478 (85.3) | 769 (14.7) |  | 13,432 (80.9) | 3162 (19.1) |  | 52,998 (78.1) | 14,819 (21.9) |  | 218,038 (79.8) | 55,161 (20.2) |  |
| **Number of children exposed to ACS** (%) | 3036 / 3665 (82.8) | 691 / 769 (89.9) | **<0.001** | 4526 / 11,836 (38.2) | 1300 / 3162 (41.1) | **0.003** | 2139 / 52,923 (4.0) | 825 / 14,819 (5.6) | **<0.001** | 589 / 217,886 (0.3) | 147 / 55,161 (0.3) | 0.88 |
| **Maternal Characteristics** | |  |  |  |  |  |  |  |  |  |  |  |
| **Maternal age at delivery (years)**, mean (SD) | 29.2 (6.3) | 28.9 (6.1) | 0.28 | 29.5 (6.2) | 29.5 (6.0) | 0.70 | 29.7 (6.0) | 29.7 (5.9) | 0.62 | 29.3 (5.8) | 29.8 (5.6) | **<0.001** |
| **Parity: Primiparous**, N (%) | 2159 / 4442 (48.6) | 349 / 768 (45.4) | 0.11 | 5873 / 13,358 (44.0) | 1314 / 3155 (41.6) | **0.02** | 19,837 / 52,761 (37.6) | 5380 / 14,794 (36.4) | **0.006** | 92,756 / 216,913 (42.8) | 22,639 / 55,102 (41.1) | **<0.001** |
| **Maternal Body Mass Index (kg/m^2^)**, N (%) |  |  | 0.36 |  |  | **0.009** |  |  | **<0.001** |  |  | **<0.001** |
| Underweight (<18.5) | 187 (4.8) | 30 (4.2) |  | 493 (4.0) | 152 (5.0) |  | 1741 (3.6) | 486 (3.4) |  | 5245 (2.6) | 1311 (2.4) |  |
| Normal weight (18.5-24.9) | 1801 (46.0) | 320 (44.3) |  | 5678 (46.5) | 1344 (44.3) |  | 21,832 (44.7) | 6251 (43.2) |  | 98,529 (48.8) | 25,601 (47.5) |  |
| Overweight (25-29.9) | 1009 (25.8) | 208 (28.8) |  | 3227 (26.4) | 788 (26.0) |  | 13,127 (26.9) | 3895 (26.9) |  | 56,661 (28.1) | 15,281 (28.3) |  |
| Obese (≥30) | 921 (23.5) | 164 (22.7) |  | 2809 (23.0) | 752 (24.8) |  | 12,136 (24.9) | 3831 (26.5) |  | 41,337 (20.5) | 11,753 (21.8) |  |
| **Maternal diabetes (any)**, N (%) | 248 / 4313 (5.8) | 60 / 754 (8.0) | **0.02** | 1072 / 13,125 (8.2) | 326 / 3140 (10.4) | **<0.001** | 3943 / 51,952 (7.6) | 1261 / 14,742 (8.6) | **<0.001** | 3214 / 213,573 (1.5) | 1093 / 54,882 (2.0) | **<0.001** |
| **Smoking at first antenatal appointment**, N (%) | 1154 / 4103 (28.1) | 205 / 726 (28.2) | 0.95 | 3192 / 12,778 (25.0) | 730 / 3072 (23.8) | 0.16 | 10,692 / 50,821 (21.0) | 2830 / 14,508 (19.5) | **<0.001** | 35,868 / 210,444 (17.0) | 7556 / 54,214 (13.9) | **<0.001** |
| **Child Characteristics** |  |  |  |  |  |  |  |  |  |  |  |  |
| **Child sex: Girl**, N (%) | 1361 / 3139 (43.4) | 330 / 769 (42.9) | 0.82 | 4715 /10,236 (46.1) | 1448 / 3162 (45.8) | 0.79 | 18,998 / 40,167 (47.3) | 7056 / 14,819 (47.6) | 0.51 | 83,831 / 170,893 (49.1) | 27,093 / 55,161 (49.1) | 0.80 |
| **Year of birth**, N(%) |  |  | **<0.001** |  |  | **<0.001** |  |  | **<0.001** |  |  | **<0.001** |
| 2011 | 803 (17.9) | 0 (0.0) |  | 2260 (16.8) | 0 (0.0) |  | 9058 (17.1) | 0 (0.0) |  | 42,031 (19.3) | 0 (0.0) |  |
| 2012 | 744 (16.6) | 0 (0.0) |  | 2321 (17.3) | 0 (0.0) |  | 9099 (17.2) | 0 (0.0) |  | 41,413 (19.0) | 0 (0.0) |  |
| 2013 | 785 (17.5) | 0 (0.0) |  | 2234 (16.6) | 0 (0.0) |  | 9032 (17.0) | 0 (0.0) |  | 39,932 (18.3) | 0 (0.0) |  |
| 2014 | 737 (16.5) | 0 (0.0) |  | 2303 (17.1) | 0 (0.0) |  | 9554 (18.0) | 0 (0.0) |  | 40,035 (18.4) | 0 (0.0) |  |
| 2015 | 470 (10.5) | 277 (36.0) |  | 1465 (10.9) | 1078 (34.1) |  | 5293 (10.0) | 4733 (31.9) |  | 19,630 (9.0) | 18,283 (33.1) |  |
| 2016 | 439 (9.8) | 286 (37.2) |  | 1280 (9.5) | 1166 (36.9) |  | 4569 (8.6) | 5759 (38.9) |  | 14,932 (6.8) | 21,600 (39.2) |  |
| 2017 | 500 (11.2) | 206 (26.8) |  | 1569 (11.7) | 918 (29.0) |  | 6393 (12.1) | 4327 (29.2) |  | 20,065 (9.2) | 15,278 (27.7) |  |
| **Gestational age at birth**, mean (SD) | 31.2 (1.6) | 31.5 (1.6) | **<0.001** | 35.4 (0.8) | 35.5 (0.8) | **<0.001** | 37.69 (0.5) | 37.66 (0.5) | **<0.001** | 39.91 (0.8) | 38.89 (0.8) | **<0.001** |
| **Birthweight (g)**, mean (SD) | 1688.3 (487.6) | 1724.9 (461.2) | 0.05 | 2614.0 (498.6) | 2640.9 (484.5) | **0.006** | 3159.4 (482.3) | 3166.0 (477.2) | 0.14 | 3552.0 (455.5) | 3580.2 (449.2) | **<0.001** |
| **Neighbourhood deprivation (SIMD deciles)**, mean (SD) | 4.5 (2.9) | 4.5 (2.7) | 0.70 | 4.6 (2.9) | 4.9 (2.8) | **<0.001** | 4.8 (2.9) | 5.0 (2.8) | **<0.001** | 5.1 (2.9) | 5.4 (2.8) | **<0.001** |

Tables S5(A-B). The association of covariates with neurodevelopmental outcomes, stratified by gestational age at birth.

Referent category shown in **[*italics]***.

Accompanying text is found in **Text S2**.

95% CI = 95% confidence intervals. ASQ-3 = Ages and Stages Questionnaire – Third Edition**.** B = Coefficient*. BMI = Body Mass Index. OR = Odds Ratio. SD = Standard deviation. SIMD = Scottish Index of Multiple Deprivation (1=most deprived decile, 10=least deprived decile).

^a^ assessed at first antenatal booking appointment.

^b^ ASQ-3 was only introduced from 2017, hence children born before 2015 did have not assessment of ASQ-3 scores.

*The B values and 95% CIs for models 1-3 can be interpreted as reflecting the mean unadjusted and adjusted group differences of ACS-exposed versus non-ACS-exposed children in the ASQ-3 neurodevelopmental scores in standard deviation units, where the mean score in standard deviation units for whole cohort is 0 and standard deviation is 1.

Table S5-A. The association of covariates with the presence of practitioner concern about neurodevelopment, stratified by gestational age at birth

| **COVARIATE** | **28-33 weeks’ gestation** | | **34-36 weeks’ gestation** | | **37-38 weeks’ gestation** | | **39-41 weeks’ gestation** | |
| --- | --- | --- | --- | --- | --- | --- | --- | --- |
|  | **OR (95% CI)** | **p value** | **OR (95% CI)** | **p value** | **OR (95% CI)** | **p value** | **OR (95% CI)** | **p value** |
| **Maternal age** | 0.86 (0.80; 0.92) | **<0.001** | 0.88 (0.84; 0.92) | **<0.001** | 0.82 (0.80; 0.83) | **<0.001** | 0.77 (0.76; 0.77) | **<0.001** |
| **Parity *[nulliparous]*** | 1.33 (1.14; 1.55) | **<0.001** | 1.30 (1.19; 1.42) | **<0.001** | 1.20 (1.15; 1.26) | **<0.001** | 1.10 (1.07; 1.12) | **<0.001** |
| **Maternal BMI *[healthy weight 18.5-24.9 kg/m^2^]***^a^ |  |  |  |  |  |  |  |  |
| Underweight (<18.5) | 1.34 (0.93; 1.93) | 0.11 | 1.42 (1.14; 1.77) | **0.002** | 1.37 (1.22; 1.54) | **<0.001** | 1.52 (1.42; 1.63) | **<0.001** |
| Overweight (25-29.9) | 0.97 (0.80; 1.19) | 0.79 | 0.98 (0.88; 1.10) | 0.71 | 1.07 (1.02; 1.13) | **0.01** | 1.09 (1.06; 1.13) | **<0.001** |
| Obese (≥ 30) | 1.24 (1.01; 1.52) | **0.04** | 1.23 (1.10; 1.38) | **<0.001** | 1.34 (1.27; 1.41) | **<0.001** | 1.41 (1.37; 1.45) | **<0.001** |
| **Maternal diabetes (any type)** | 1.35 (0.99; 1.83) | 0.06 | 1.05 (0.90; 1.22) | 0.56 | 1.12 (1.03; 1.21) | **0.01** | 1.15 (1.05; 1.25) | **0.003** |
| **Smoking** | 1.89 (1.59; 2.25) | **<0.001** | 1.97 (1.79; 2.18) | **<0.001** | 1.96 (1.87; 2.06) | **<0.001** | 2.00 (1.94; 2.06) | **<0.001** |
| **Child sex** | 0.63 (0.54; 0.74) | **<0.001** | 0.49 (0.44; 0.53) | **<0.001** | 0.49 (0.47; 0.51) | **<0.001** | 0.45 (0.44; 0.46) | **<0.001** |
| **Year of birth *[2017]*** |  |  |  |  |  |  |  |  |
| 2011 | 1.26 (0.91; 1.74) | 0.17 | 1.28 (1.06; 1.54) | **0.01** | 1.34 (1.23; 1.46) | **<0.001** | 1.59 (1.51; 1.68) | **<0.001** |
| 2012 | 1.21 (0.87; 1.69) | 0.25 | 1.39 (1.16; 1.67) | **<0.001** | 1.31 (1.20; 1.43) | **<0.001** | 1.57 (1.49; 1.65) | **<0.001** |
| 2013 | 1.24 (0.90; 1.72) | 0.19 | 1.43 (1.19; 1.72) | **<0.001** | 1.26 (1.15; 1.37) | **<0.001** | 1.48 (1.40; 1.55) | **<0.001** |
| 2014 | 1.17 (0.85; 1.62) | 0.34 | 1.29 (1.07; 1.55) | **0.006** | 1.22 (1.12; 1.33) | **<0.001** | 1.38 (1.31; 1.45) | **<0.001** |
| 2015 | 0.95 (0.68; 1.31) | 0.74 | 1.19 (1.00; 1.43) | 0.06 | 1.04 (0.95; 1.13) | 0.40 | 1.17 (1.11; 1.23) | **<0.001** |
| 2016 | 0.95 (0.68; 1.33) | 0.76 | 0.92 (0.76; 1.11) | 0.36 | 0.99 (0.90; 1.08) | 0.76 | 1.07 (1.01; 1.13) | **0.02** |
| **Child age at review** | 1.03 (0.95; 1.10) | 0.52 | 1.11 (1.07; 1.16) | **<0.001** | 1.08 (1.06; 1.10) | **<0.001** | 1.10 (1.09; 1.12) | **<0.001** |
| **Neighbourhood deprivation (SIMD deciles)** | 0.71 (0.66; 0.78) | **<0.001** | 0.70 (0.66; 0.73) | **<0.001** | 0.68 (0.67; 0.70) | **<0.001** | 0.69 (0.68; 0.70) | **<0.001** |

Table S5-B. The association of covariates with the ASQ-3 neurodevelopment score, stratified by gestational age at birth.

| **COVARIATE** | **28-33 weeks’ gestation** | | **34-36 weeks’ gestation** | | **37-38 weeks’ gestation** | | **39-41 weeks’ gestation** | |
| --- | --- | --- | --- | --- | --- | --- | --- | --- |
|  | **B (95% CI)** | **p value** | **B (95% CI)** | **p value** | **B (95% CI)** | **p value** | **B (95% CI)** | **p value** |
| **Maternal age** | -0.06 (-0.13; 0.01) | 0.1 | 0.07 (0.03; 0.10) | **<0.001** | 0.07 (0.05; 0.09) | **<0.001** | 0.06 (0.05; 0.07) | **<0.001** |
| **Parity *[nulliparous]*** | -0.13 (-0.27; 0.02) | 0.09 | 0.004 (-0.07; 0.08) | 0.92 | -0.01 (-0.05; 0.02) | 0.55 | 0.03 (0.01; 0.05) | **0.001** |
| **Maternal BMI *[healthy weight 18.5-24.9 kg/m^2^]*** ^a^ |  |  |  |  |  |  |  |  |
| Underweight (<18.5) | 0.14 (-0.26; 0.53) | 0.49 | -0.14 (-0.31; 0.03) | 0.11 | -0.08 (-0.17; 0.02) | 0.11 | -0.10 (-0.16; -0.05) | **<0.001** |
| Overweight (25-29.9) | 0.02 (-0.17; 0.20) | 0.86 | -0.05 (-0.13; 0.05) | 0.33 | -0.04 (-0.08; 0.01) | 0.08 | -0.02 (-0.04; -0.002) | **0.03** |
| Obese (≥ 30) | -0.12 (-0.31; 0.08) | 0.24 | -0.07 (-0.16; 0.02) | 0.14 | -0.11 (-0.15; -0.07) | **<0.001** | -0.10 (-0.12; -0.08) | **<0.001** |
| **Maternal diabetes (any type)** | -0.06 (-0.33; 0.21) | 0.68 | 0.001 (-0.12; 0.12) | 0.99 | -0.09 (-0.15; -0.03) | **0.005** | -0.17 (-0.23; -0.11) | **<0.001** |
| **Smoking** | -0.17 (-0.33; -0.01) | **0.04** | -0.26 (-0.35; -0.18) | **<0.001** | -0.18 (-0.22; -0.13) | **<0.001** | -0.20 (-0.23; -0.18) | **<0.001** |
| **Child sex** | 0.27 (0.13; 0.42) | **<0.001** | 0.48 (0.41; 0.55) | **<0.001** | 0.47 (0.43; 0.50) | **<0.001** | 0.49 (0.48; 0.51) | **<0.001** |
| **Year of birth *[2017]*** |  |  |  |  |  |  |  |  |
| 2011 | NA^b^ |  | NA^b^ |  | NA^b^ |  | NA^b^ |  |
| 2012 | NA^b^ |  | NA^b^ |  | NA^b^ |  | NA^b^ |  |
| 2013 | NA^b^ |  | NA^b^ |  | NA^b^ |  | NA^b^ |  |
| 2014 | NA^b^ |  | NA^b^ |  | NA^b^ |  | NA^b^ |  |
| 2015 | -0.01 (-0.16; 0.14) | 0.87 | -0.09 (-0.16; -0.01) | **0.03** | -0.06 (-0.09; -0.02) | **0.002** | -0.05 (-0.06; -0.03) | **<0.001** |
| 2016 | 0.08 (-0.07; 0.23) | 0.31 | 0.06 (-0.02; 0.13) | 0.14 | 0.01 (-0.03; 0.04) | 0.76 | -0.003 (-0.02; 0.01) | 0.73 |
| **Child age at review** | 0.16 (0.07; 0.24) | **<0.001** | 0.12 (0.08; 0.16) | **<0.001** | 0.13 (0.11; 0.15) | **<0.001** | 0.13 (0.12; 0.14) | **<0.001** |
| **Neighbourhood deprivation (SIMD deciles)** | 0.07 (-0.01; 0.15) | 0.07 | 0.08 (0.04; 0.12) | **<0.001** | 0.08 (0.06; 0.10) | **<0.001** | 0.08 (0.07; 0.09) | **<0.001** |

Table S6(A-B). Sensitivity analyses restricted to children who were born as a result of mothers’ first (or only) index pregnancies within the cohort

Table S6-A. The association of ACS exposure with practitioner concerns about neurodevelopment, stratified by gestational age, limited to children who were born as a result of mothers’ first (or only) index pregnancies within the cohort

| **Gestational age at birth**  **(weeks’ gestation)** | **Presence of practitioner concerns about neurodevelopment** | | | | | | | | |
| --- | --- | --- | --- | --- | --- | --- | --- | --- | --- |
|  | **N of children with concern / N of children with outcome data** | | | **Model 1**^a^  Unadjusted | | **Model 2**^b^  Minimally adjusted | | **Model 3**^c^  Fully adjusted | |
|  | **Total** | **Non-ACS-exposed** | **ACS-exposed** | **OR (95% CI)** | **p value** | **OR (95% CI)** | **p value** | **OR (95% CI)** | **p value** |
| **28-33** | 820 / 2622 (31.3) | 115 / 301 (38.2) | 705 / 2321 (30.4) | **0.71 (0.55; 0.91)** | **0.006** | **0.70 (0.54; 0.90)** | **0.005** | **0.76 (0.58; 0.98)** | **0.04** |
| **34-36** | 2201 / 9301 (23.7) | 1303 / 5695 (22.9) | 898 / 3606 (24.9) | **1.12 (1.01; 1.23)** | **0.03** | **1.11 (1.01; 1.23)** | **0.04** | 1.09 (0.98; 1.21) | 0.11 |
| **37-38** | 8554 / 40,452 (21.1) | 8250 / 38,906 (21.1) | 304 / 1546 (19.7) | 0.91 (0.80; 1.03) | 0.15 | 0.93 (0.82; 1.06) | 0.29 | 0.95 (0.83; 1.08) | 0.42 |
| **39-41** | 29,720 / 171,943 (17.3) | 29,626 / 171,479 (17.3) | 94 / 464 (20.3) | 1.22 (0.97; 1.53) | 0.09 | 1.23 (0.98; 1.54) | 0.08 | 1.10 (0.87; 1.38) | 0.45 |

ACS = antenatal corticosteroids. CI = confidence intervals. N = number. OR = odds ratio.

^a^ Model 1 is unadjusted.

^b^ Model 2 adjusts for child sex and child age at review.

^c^ Model 3 adjusts for child sex, child age at review and maternal age, maternal BMI, smoking status at first antenatal appointment, parity, maternal diabetes, year of birth and neighbourhood deprivation.

Table S6-B. The association of ACS exposure with ASQ-3 neurodevelopment score, stratified by gestational age, limited to children who were born as a result of mothers’ first (or only) index pregnancies within the cohort

| **Gestational age at birth**  **(weeks’ gestation)** | **ASQ-3 neurodevelopment score** | | | | | | | | | |
| --- | --- | --- | --- | --- | --- | --- | --- | --- | --- | --- |
|  | **Number of children with ASQ-3 neurodevelopment score** | | | | **Model 1**^a^  Unadjusted | | **Model 2**^b^  Minimally adjusted | | **Model 3**^c^  Fully adjusted | |
|  | **Total** | **Non-ACS-exposed** | **ACS-exposed** | **B (95% CI)** | | **p value** | **B (95% CI)** | **p value** | **B (95% CI)** | **p value** |
| **28-33** | 562 | 52 | 510 | 0.15 (-0.14; 0.43) | | 0.32 | 0.10 (-0.18; 0.39) | 0.47 | 0.07 (-0.21; 0.36) | 0.62 |
| **34-36** | 2225 | 1310 | 915 | **0.11 (0.02; 0.19)** | | **0.01** | **0.09 (0.004; 0.17)** | **0.04** | **0.09 (0.004; 0.17)** | **0.04** |
| **37-38** | 9685 | 9195 | 490 | -0.06 (-0.15; 0.03) | | 0.2 | -0.05 (-0.14; 0.04) | 0.25 | -0.04 (-0.13; 0.05) | 0.34 |
| **39-41** | 36,994 | 36,895 | 99 | -0.11 (-0.30; 0.09) | | 0.28 | -0.12 (-0.31; 0.07) | 0.22 | -0.10 (-0.28; 0.09) | 0.3 |

ACS = antenatal corticosteroids. ASQ-3 = Ages and Stages Questionnaire Third Edition. B = Unstandardised regression coefficient, with the outcome variable expressed in standard deviation units*. CI = confidence interval.

^a^ Model 1 is unadjusted.

^b^ Model 2 for child sex and child age at review.

^c^ Model 3 adjusts for child sex, child age at review and maternal age, maternal BMI, smoking status at first antenatal appointment, parity, maternal diabetes, year of birth and neighbourhood deprivation.

*The B values and 95% CIs for models 1-3 can be interpreted as reflecting the mean unadjusted and adjusted group differences of ACS-exposed versus non-ACS-exposed children in the ASQ-3 neurodevelopmental scores in standard deviation units, where the mean score in standard deviation units for whole cohort is 0 and standard deviation is 1.

**References for Tables S1-S6**

1. RCOG. Green-top Guideline No. 7: Antenatal corticosteroids to reduce neonatal morbidity and mortality. Royal College of Obstetricians and Gynaecologists; 2010.

2. NICE. NICE Guideline NG25: Preterm labour and birth. National Institute for Health and Care Excellence; 2015.

3. Squires J, Bricker D. *Ages & Stages Questionnaires®, Third Edition (ASQ®-3): A Parent-Completed Child Monitoring System.* 2009. <https://agesandstages.com/>

4. *Early Child Development Statistics 2021/22 - Technical Report*. 2023. <https://publichealthscotland.scot/media/19171/2023-04-25-early-child-development-technical-report.pdf>

5. Lahti-Pulkkinen M, Mina TH, Riha RL, et al. Maternal antenatal daytime sleepiness and child neuropsychiatric and neurocognitive development. *Psychological Medicine*. Sep 2019;49(12):2081-2090. doi:10.1017/s003329171800291x
