# Supplementary material for: Associations of Antenatal Corticosteroids With Neurodevelopment in Children Aged 27–30 Months: A Population‐Based Cohort Study
Source: BJOG. 2025 Feb 19;132(7):902–15. doi: 10.1111/1471-0528.18101 (PMC12051224; doi:10.1111/1471-0528.18101)
Supplement: Supplementary file 3 — Text S1‐S3. [file BJO-132-902-s003.docx]

# Supporting information (Text)

Text S1. STROBE checklist.

|  | | Item No | Recommendation |
| --- | --- | --- | --- |
| **Title and abstract** | | 1 | (*a*) Indicate the study’s design with a commonly used term in the title or the abstract (Title page; Abstract – Methods) |
|  |  |  | (*b*) Provide in the abstract an informative and balanced summary of what was done and what was found (Abstract – Methods, Results, Discussion) |
| Introduction | | | |
| Background/rationale | | 2 | Explain the scientific background and rationale for the investigation being reported (Introduction – paragraphs 1, 2, 3, 4) |
| Objectives | | 3 | State specific objectives, including any prespecified hypotheses (Introduction – paragraph 4) |
| Methods | | | |
| Study design | | 4 | Present key elements of study design early in the paper (Methods – Study population) |
| Setting | | 5 | Describe the setting, locations, and relevant dates, including periods of recruitment, exposure, follow-up, and data collection (Methods – Study population, ACS exposure, Outcomes) |
| Participants | | 6 | (*a*) Give the eligibility criteria, and the sources and methods of selection of participants. Describe methods of follow-up (Methods – Study population) |
|  |  |  | (*b*) For matched studies, give matching criteria and number of exposed and unexposed *– Not applicable.* |
| Variables | | 7 | Clearly define all outcomes, exposures, predictors, potential confounders, and effect modifiers. Give diagnostic criteria, if applicable (Methods – ACS exposure, Outcomes, Covariates and possible confounders; Supporting information – Table S2, Table S3) |
| Data sources/ measurement | | 8* | For each variable of interest, give sources of data and details of methods of assessment (measurement). Describe comparability of assessment methods if there is more than one group (Methods – Study population, ACS exposure, Outcomes; Supporting information – Table S2, Table S3) |
| Bias | | 9 | Describe any efforts to address potential sources of bias (Statistical analysis – paragraphs 2,4) |
| Study size | | 10 | Explain how the study size was arrived at (Methods – Study population) |
| Quantitative variables | | 11 | Explain how quantitative variables were handled in the analyses. If applicable, describe which groupings were chosen and why (Methods – Outcomes; Statistical analysis – paragraphs 1, 4; Supporting information – Table S2) |
| Statistical methods | | 12 | (*a*) Describe all statistical methods, including those used to control for confounding (Statistical analysis – paragraphs 1-4) |
|  |  |  | (*b*) Describe any methods used to examine subgroups and interactions (Statistical analysis – paragraph 1,4) |
|  |  |  | (*c*) Explain how missing data were addressed (Methods – Study population, Outcomes; Statistical analysis – paragraph 2; Supporting information – Table S2) |
|  |  |  | (d) Cohort study—If applicable, explain how loss to follow-up was addressed. (Methods – Study population; Statistical analysis – paragraph 2) |
|  |  |  | (*e*) Describe any sensitivity analyses (Methods – Study population; Supporting information – Table S4) |
| Results | | | |
| Participants | 13* | (a) Report numbers of individuals at each stage of study—eg numbers potentially eligible, examined for eligibility, confirmed eligible, included in the study, completing follow-up, and analysed (Results - Figure 1, paragraph 1, Tables 1-4; Supporting information – Table S4) | |
|  |  | (b) Give reasons for non-participation at each stage (Methods – Study population, Figure 1, ACS exposure, Outcomes; Supporting information – Table S2) | |
|  |  | (c) Consider use of a flow diagram (Results – Figure 1) | |
| Descriptive data | 14* | (a) Give characteristics of study participants (eg demographic, clinical, social) and information on exposures and potential confounders (Results - Demographics, Children born at 28-33 weeks’ gestation, Children born at 34-36 weeks’ gestation, Children born at 37-38 weeks’ gestation, Children born at 39-41 weeks’ gestation, Tables 1-4) | |
|  |  | (b) Indicate number of participants with missing data for each variable of interest (Tables 1-2) | |
|  |  | (c) *Cohort study*—Summarise follow-up time (eg, average and total amount) (Results - Demographics, Table 2) | |
| Outcome data | 15* | *Cohort study*—Report numbers of outcome events or summary measures over time (Results - Children born at 28-33 weeks’ gestation, Children born at 34-36 weeks’ gestation, Children born at 37-38 weeks’ gestation, Children born at 39-41 weeks’ gestation, Tables 1-4) | |
| Main results | 16 | (*a*) Give unadjusted estimates and, if applicable, confounder-adjusted estimates and their precision (eg, 95% confidence interval). Make clear which confounders were adjusted for and why they were included (Results - Table 3, Table 4; Methods – Covariates and possible confounders; Supporting information – Figure S1) | |
|  |  | (*b*) Report category boundaries when continuous variables were categorized (Methods – Covariates and possible confounders; Table 1-2) | |
|  |  | (*c*) If relevant, consider translating estimates of relative risk into absolute risk for a meaningful time period | |
| Other analyses | 17 | Report other analyses done—eg analyses of subgroups and interactions, and sensitivity analyses (Results, Supporting information – Text S2, Table S4, Tables S5(A-B)) | |
| Discussion | | | |
| Key results | 18 | Summarise key results with reference to study objectives (Discussion – Main Findings, Conclusion) | |
| Limitations | 19 | Discuss limitations of the study, taking into account sources of potential bias or imprecision. Discuss both direction and magnitude of any potential bias (Discussion – Strengths and Limitations) | |
| Interpretation | 20 | Give a cautious overall interpretation of results considering objectives, limitations, multiplicity of analyses, results from similar studies, and other relevant evidence (Discussion – Interpretation) | |
| Generalisability | 21 | Discuss the generalisability (external validity) of the study results (Discussion – Interpretation, Strengths and Limitations) | |
| Other information | | | |
| Funding | 22 | Give the source of funding and the role of the funders for the present study and, if applicable, for the original study on which the present article is based (Funding) | |

Text S2. Gestation-specific associations of covariates with ACS exposure and with neurodevelopmental outcomes

The following text accompanies **Table 1**, **Table S5-A** and **Table** **S5-B.**

**Children born at 28-33 weeks’ gestation**

The incidences of maternal smoking and neighbourhood deprivation were lower in the ACS-exposed group than the non-ACS-exposed group. Compared to non-ACS-exposed children at this gestation, ACS-exposed children were older at review.

Multiparity, maternal obesity, antenatal smoking and neighbourhood deprivation were associated with increased odds of practitioner concerns about neurodevelopment. Older maternal age and female child sex were associated with reduced odds of practitioner concerns about neurodevelopment. Child age at review and female child sex were associated positively with ASQ-3 neurodevelopment scores, while antenatal smoking was associated negatively with ASQ-3 neurodevelopment scores.

**Children born at 34-36 weeks’ gestation**

There was a higher incidence of maternal diabetes and mothers who were overweight or obese in the ACS-exposed group compared to the non-ACS-exposed group. Mean maternal age was higher in ACS-exposed children at this gestation compared to unexposed children, as was birth in 2013, 2014 and 2015. Compared to non-ACS-exposed children at this gestation, ACS-exposed children were older at review, lived in less deprived neighbourhoods, and had been born at earlier gestations, with lower birthweights.

Multiparity, an underweight or obese maternal BMI, antenatal smoking, birth between 2011 and 2014, neighbourhood deprivation and older age at review were associated with increased odds of practitioner concerns about neurodevelopment. Older maternal age and female child sex were associated with reduced odds of practitioner concerns about neurodevelopment and associated positively with ASQ-3 neurodevelopment scores. Older children had higher ASQ-3 neurodevelopment scores, while antenatal smoking, neighbourhood deprivation and birth in 2015 were associated negatively with ASQ-3 neurodevelopment scores.

**Children born at 37-38 weeks’ gestation**

The incidences of maternal diabetes, maternal obesity, and birth during 2014 to 2017 were higher in ACS-exposed children compared to non-ACS-exposed children. Mean maternal age, gestational age at birth, birthweight, age at time of review and the incidence of neighbourhood deprivation were lower in children at this gestation who were ACS-exposed compared to those who were unexposed, and mothers were less often nulliparous.

Multiparity, maternal diabetes, antenatal smoking, antenatal BMI outside the healthy range (18.5-24.9 kg/m^2^), birth between 2011 and 2014, neighbourhood deprivation, and older age at review were associated with increased odds of practitioner concerns about neurodevelopment. Older maternal age and female child sex were associated with reduced odds of practitioner concerns about neurodevelopment. Older maternal age, older age at review, and female child sex were associated positively with ASQ-3 neurodevelopment scores, and maternal obesity, maternal diabetes, antenatal smoking, birth in 2015 and neighbourhood deprivation were associated negatively with ASQ-3 neurodevelopment scores.

**Children born at 39-41 weeks’ gestation**

The incidences of multiparity, antenatal smoking, maternal diabetes and neighbourhood deprivation were higher in the ACS-exposed group than the non-ACS-exposed group. Compared to non-ACS-exposed children at this gestation, ACS-exposed children had older mothers, were more often born between 2013 and 2017. ACS-exposed children at this gestation had lower mean gestational age at birth and birthweights compared to unexposed children.

Multiparity, antenatal smoking, maternal diabetes, antenatal BMI outside the healthy range (18.5-24.9 kg/m^2^), neighbourhood deprivation and older age at review were associated with increased odds of practitioner concerns about neurodevelopment. Older maternal age, birth in 2017 and female child sex were associated with reduced odds of practitioner concerns. Older maternal age, multiparity, female child sex and older age at review were associated positively with ASQ-3 neurodevelopment score. Maternal diabetes, antenatal smoking, antenatal BMI outside the healthy range (18.5-24.9 kg/m^2^), birth in 2015 and neighbourhood deprivation were associated negatively with ASQ-3 neurodevelopment score.

Text S3. Attrition analyses comparing children included and excluded from statistical analyses of specific neurodevelopmental outcomes

The following text accompanies **Tables S4-B** and **S4-C.**

**Attrition analyses of children excluded from analyses of practitioner concerns about neurodevelopment**

On comparing children excluded from the practitioner-identified concern analyses versus children included in these analyses, we found that in all gestational age groups, children excluded from the analyses of practitioner concerns about neurodevelopment were more often born in the later years of the cohort, from multiparous pregnancies, and they had lower birthweights, compared to children included in the analyses of practitioner concerns about neurodevelopment.

The mothers of the excluded children born at 39-41 weeks’ gestation were older than the mothers of children in this gestational age category who were included in analyses of practitioner-identified neurodevelopmental concerns, and they more often had diabetes and were less often obese than the mothers of included children. The neighbourhood deprivation level was lower in the excluded children born at 34-36 weeks’ gestation compared with the included children born at 34-36 weeks’ gestation, while the level of neighbourhood deprivation of the excluded children born at 39-41 weeks’ gestation was higher than that of the included children born in the same gestational age category. Within the gestational age groups 28-33, 34-36 and 39-41 weeks’ gestation, the excluded children had lower gestational age at birth compared to the included children. The excluded children born at 28-33 weeks’ gestation had less often been exposed to ACS than the included children born in the same gestational age group.

**Attrition analyses of children excluded from analyses of ASQ-3 neurodevelopment scores**

We found that in all gestational age groups, children excluded from the ASQ-3 analyses (because of missing ASQ-3 data in any ASQ-3 domain) were more often born in the earlier years of the cohort (as expected, ASQ-3 data were only available for children born from 2015 onwards). The proportion of the mothers who had diabetes was smaller in the excluded children compared to mothers of children included in the ASQ-3 analyses. Furthermore, in children born at 28-33, 34-36 and 37-38 weeks’ gestation, a smaller proportion of children excluded had been exposed to ACS compared to the children included. In children born at 34-36, 37-38 and 39-41 weeks’ gestation, the excluded children more often had a primiparous mother, who were less often obese at the first antenatal appointment. Additionally, in children born at 37-38 and at 39-41 weeks’ gestation, the mothers of the excluded children smoked more often at first antenatal appointment than the mothers of the included children, and in children born at 39-41 weeks’ gestation, mothers of excluded children were younger than mothers of included children. Finally, gestational age at birth was associated with attrition status in all gestational age groups, with opposing patterns in children born at 28-33 and 34-36 weeks’ gestation (excluded children were born earlier than those included) than children born at 37-38 and 39-41 weeks’ gestation (included children were born earlier than those excluded).
